# Optimizing Public Health Preparedness for Highly Infectious Diseases in Central Vietnam

**DOI:** 10.3390/diagnostics12092047

**Published:** 2022-08-24

**Authors:** Amanullah Zadran, An V. D. Ho, Layma Zadran, Irene J. Ventura Curiel, Tang-Tung Pham, Duong Thi Bich Thuan, Gerald J. Kost

**Affiliations:** 1 Point-of-Care Testing Center for Teaching and Research (POCT CTR^TM^) Pathology and Laboratory Medicine, School of Medicine, University of California, Davis, CA 95616, USA; 2 Department of Orthopedics and Rehabilitation, University of Medicine and Pharmacy at Ho Chi Minh City, Ho Chi Minh City 70000, Vietnam; 3 Faculty of Medicine, Phan Chau Trinh University, No 9 Nguyen Gia Thieu St, Dien Ban Ward, Dien Ngoc District, Danang City 550000, Vietnam; 4 College of Health Sciences, VinUniversity, Vinhomes Ocean Park, Gia Lam District, Hanoi 100000, Vietnam

**Keywords:** infectious disease testing, public health preparedness, point-of-care testing (POCT), molecular diagnostics, therapeutic turnaround time (TTAT), acute medical challenges, geographic information systems, antimicrobial stewardship

## Abstract

Our primary objectives were (a) to determine the need for and the availability of point-of-care testing (POCT) for infectious diseases and (b) to recommend point-of-care testing strategies and Spatial Care Paths^TM^ (SCPs) that enhance public health preparedness in the regional districts of Thua Thien Hue Province (TTHP), Central Vietnam, where we conducted field surveys. Medical professionals in seven community health centers (CHCs), seven district hospitals (DHs) and one provincial hospital (PH) participated. Survey questions (English and Vietnamese) determined the status of diagnostic testing capabilities for infectious diseases and other acute medical challenges in TTHP. Infectious disease testing was limited: six of seven CHCs (86%) lacked infectious disease tests. One CHC (14%, 1/7) had two forms of diagnostic tests available for the detection of malaria. All CHCs lacked adequate microbiology laboratories. District hospitals had few diagnostic tests for infectious diseases (tuberculosis and syphilis), blood culture (29%, 2/7), and pathogen culture (57%, 4/7) available. The PH had broader diagnostic testing capabilities but lacked preparedness for highly infectious disease threats (e.g., Ebola, MERS-CoV, SARS, Zika, and monkeypox). All sites reported having COVID-19 rapid antigen tests; COVID-19 RT-PCR tests were limited to higher-tier hospitals. We conclude that infectious disease diagnostic testing should be improved and POC tests must be supplied near patients’ homes and in primary care settings for the early detection of infected individuals and the mitigation of the spread of new COVID-19 variants and other highly infectious diseases.

## 1. Introduction

The global emergence of highly infectious disease threats, such as the novel coronavirus disease 2019 (COVID-19), Ebola virus disease (EVD), Middle East respiratory syndrome coronavirus (MERS-CoV), severe acute respiratory syndrome (SARS), avian influenza, tuberculosis (TB), and antimicrobial resistance (AMR), pose significant risks to public health in the Asia Pacific region and to the region’s capacity to respond to these threats [1]. In 2017, there were 105,733 cases reported of TB in Vietnam, 26% of which were diagnosed by WHO-recommended rapid diagnostics tests [2]. Now in Vietnam, AMR rates have become among the highest in Asia, with multidrug-resistant (MDR) infections developing in TB, influenza, and malaria, attributed in part to misdiagnosis and more fundamentally to the increase in prescriptions and the over-the-counter use of antibiotics [3].

Despite an increase in Vietnam’s health expenditure per capita from 2006 (USD $42) to 2019 (USD $181), the poor, ethnic minorities, and those living in remote areas are at risk of public health threats [4,5,6,7,8,9]. Public health emergency preparedness for highly infectious diseases is crucial in enhancing community resilience to health threats [1]. A broader application of point-of-care (POC) pathogen detection would better characterize the ecology and epidemiology of infectious diseases and their impact in Central Vietnam. Hence, the objectives of this article are to report the status of infectious disease diagnostic testing capabilities and point-of-care testing (POCT) and to recommend healthcare strategies, point-of-care technologies, and the use of Spatial Care Paths^TM^ (SCPs) to enhance public health preparedness in the regional districts of Thua Thien Hue Province (TTHP), Central Vietnam. Vietnam can play an important role by implementing POCT to meet the serious deficit in public health emergency preparedness for infectious disease outbreaks, especially in neglected rural communities. Point-of-care testing can strengthen Vietnam’s current public health framework, disease surveillance capacity, and timely response to prevent the risks of transmission for infectious diseases [10,11,12].

## 2. Materials and Methods

### 2.1. Hospital Levels

**Figure 1:**
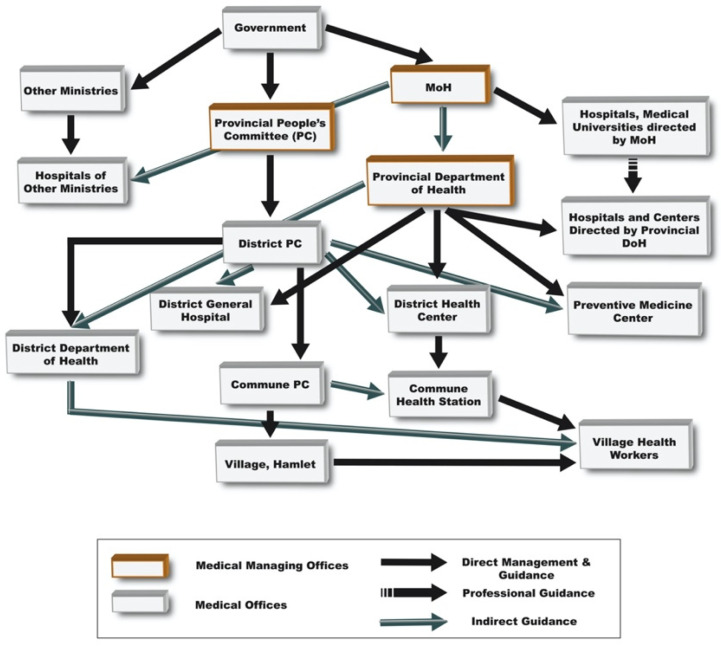
Management and funding of health facilities in Vietnam.

The Vietnamese hospitals are classified by “levels”. The most common are Level 4 hospitals, also known as community health centers (CHCs), which are equivalent to primary care centers [13]. There are several CHCs in any given district and these centers are generally ill-equipped and poorly funded. Figure 1 illustrates management, guidance, and funding from the Vietnamese Ministry of Health (MoH) to health facilities. Level 3 hospitals, commonly known as district hospitals (DHs), one per district, are typically better equipped and funded than Level 4 hospitals. There may be one or more Level 2 provincial hospitals (PHs) per province. There is one PH in TTHP, which is known as Hue University Hospital (HUH), which is even better equipped and funded. There are three Level 1 hospitals in Vietnam. One Level 1 hospital, Hue Central Hospital (HCH), is within close proximity of HUH. The administration of Level 1 hospitals is tightly associated with the central government. We did not survey the Level 1 hospital in TTHP because our focus was on rural healthcare. Hue Central Hospital infectious disease testing capability was determined through insightful dialogues throughout the field survey (see Acknowledgments) and through analyzing government directives.

### 2.2. Healthcare Insurance

Vietnamese healthcare insurance policies impose a financial deterrent on patients directly visiting downstream tertiary hospitals (Level 1 or Level 2) for non-life-threatening emergency cases. In order to avoid financial liability, a patient must first visit a Level 4 hospital in a non-life-threatening emergency case before being referred to a tertiary hospital for treatment and care.

In a *non-life-threatening emergency* case, if a patient bypasses Level 4 and Level 3 hospitals for a higher-tier tertiary hospital (Level 1 or Level 2 hospital), health insurance plans will reimburse 40% (Level 1) to 60% (Level 2) for inpatient services. This percentage is expected go up to 100% in 2021 for Level 2 hospitals and remain the same for Level 1 hospitals [14]. In *life-threatening emergency cases*, insurance plans reimburse 100% for a visit to a Level 1 or Level 2 hospital.

As of November 2015, the residents of TTHP are allowed to bypass Level 4 hospitals to visit Level 3 hospitals regardless of their perceived illness severity for inpatient and outpatient services [15]. However, this policy applies to residents in a specific province. A traveler from a neighboring province intending to visit Hue Central Hospital (1 out of 3 Level 1 hospitals in the country) or Hue University Hospital will be required to first visit a Level 4 hospital, then a Level 3 hospital, and onwards in a stepwise fashion.

Care shifted upstream to the site of the patient can conserve financial resources and time and spare lives, especially when an outbreak and epidemic has the potential to affect every hospital level.

In addition, bypassing Level 4 hospitals is common and people frequently seek care at higher-tier tertiary hospitals (Level 1 and Level 2) to get treatment for diseases that could be managed by Level 3 and Level 4 hospitals. Hence, bypassing lower-level hospitals increases the burden on higher-tier facilities, contributing to overcrowding [16].

### 2.3. Survey Scope

Thua Thien Hue Province shares its western border with Laos, its northern border with Quang Tri, its southern border with Quang Nam and Da Nang, and its eastern border with East Sea/South China Sea. Thua Thien Hue Province is divided into 9 districts and further subdivided into 8 commune-level towns, 105 communes, and 39 wards. Each district is designated a DH and several CHCs. In the center of TTHP is Hue City, the location of HUH, the PH. The survey sites were conducted in four geographical regions in TTHP: coastal, mountain, plain, and city. Field survey sites included 7 CHCs, 7 DHs, and 1 HUH.

We conducted field research on-site for 1 month using a needs assessment questionnaire that investigated the availability of POC cardiac biomarker testing for the diagnosis of AMI [17], public health challenges, molecular technology use and test clusters for infectious diseases, outbreak preparedness, sepsis, diabetes mellitus [18], and laboratory support for critically ill patients in isolation and intensive care units. The field survey was approved by the Hue University of Medicine and Pharmacy Ethics Committee, Thua Thien Hue, Vietnam (approval number: 1716/SYT-NVT).

We primarily used two survey tools: (a) a short form for community health center interviews and (b) a long form for assessing the provincial and district hospitals’ laboratory diagnostic testing capabilities and available near-bedside testing [17,18]. We arbitrarily provided (c) an infectious disease questionnaire to a community health center, the district hospital in Quang Dien, a convenient center for incoming travelers to the coastal district, and HUH. Forms were provided in both English and Vietnamese (see supplementary materials).

The responses received from each interview were obtained verbally in Vietnamese and translated to English with the assistance of medical students from Hue University of Medicine and Pharmacy (HUMP). Survey responses were recorded from several interviews with hospital administration, physicians, nurses, and clinical laboratory staff who supported the operations of the hospital. The findings reported here are primarily based on a POC needs assessment for infectious diseases and an analysis of policy documents conducted between July 2017 and mid-2022. Documents include updates on disease situations, technical guidelines, standards, and recommendations. Supplementary data collection and information were acquired after the field study through email dialogues with coauthors in Vietnam through mid-2022.

### 2.4. Needs Assessment: Infectious Disease Testing

The needs assessment survey in Central Vietnam determined the availability of departmental facilities, staff, critical care monitoring, referral sites, public health readiness, and diagnostic testing available to clinicians to enhance clinical decision making in patients with potential highly infectious diseases in TTHP. Thua Thien Hue Province is a representation of healthcare advances and decision making in Central Vietnam due to the single medical university hospital in the central region. The provincial hospital provides healthcare to locals as well as travelers from neighboring provinces who seek medical attention. The needs assessment survey describes deficiencies and provides areas that need the implementation of POCT to enhance public health resilience regionally.

### 2.5. Data Representation

Demographic data are aggregated by mean (frequency) and standard deviation (SD) (see Table 1). Table 2, Table 3, Table 4, Table 5 and Table 6 showcase the frequency of responses received by hospital level (Levels 2, 3, and 4). Table 7 and Table 8 report the infectious disease questionnaire findings from the coastal province of Quang Dien and HUH. Table 9 presents government directives available for highly infectious disease agents. The percentage of available diagnostic tests by hospital level is shown in Table 10.

### 2.6. Geographic Information System, SWNs, and SCPs

A Spatial Care Path^TM^ (SCP) is the most efficient route taken by a patient to receive definitive care in the small-world network (SWN) of the healthcare delivery system [19]. SWNs are geographic relationships between patients, healthcare resources, and facilities that provide care [20]. SCPs can shift diagnosis upstream in TTHP by utilizing the temporal and geospatial dimensions that characterize the SWN.

Political administration boundaries obtained from the Database of Global Administrative Areas were used to represent the TTHP of Vietnam [21]. Health resource facilities’ geographic coordinates were obtained from the Family Medicine Department at Hue University Hospital, Google Maps^®^, and Places Map^®^. Health facilities that could not be located were called for their physical addresses. Nearby landmarks were used to approximate the locations of health facilities on unnamed roads.

All coordinate data were projected using WGS84 EPSG 4326 in Quantum *Geographic Information System* (QGIS) *Las Palmas* version 2.18, a free open source GIS software package. Travel times in the SWN were analyzed using *OSM tools* plugin version 2.1 and *OpenLayers* plugin version 1.4.8 in QGIS.

## 3. Results

### 3.1. Health Statistics

In 2018, the General Statistics Office of Vietnam reported 125,200 cases of hemorrhagic fever (16 died); 127,400 cases of hand, foot, and mouth disease; 628 cases of typhoid; 760 cases of virus encephalitis (21 died); 33 cases of meningococcal disease (2 died); 673 cases of whooping cough (2 died); 77 cases of streptococcus suis infections in humans (6 died); and 2 cases of Zika virus infections [22]. As of June 2022, the MoH reported 43,088 people have died from the COVID-19 pandemic [23].

Tuberculosis and human immunodeficiency virus (HIV)/acquired immunodeficiency syndrome (AIDS) are the leading causes of death in Vietnam [24]. In December 2018, the number of individuals infected with HIV was over 208,800 and 94,900 of those cases turned into AIDS. The number of individuals that died of HIV/AIDS countrywide was 98,160 [22]. Roughly, 130,000 people are diagnosed with TB in Vietnam annually [25], of which 7000 are infected with TB and HIV. Nearly 5000 people have multidrug-resistant TB, and approximately 300 individuals have extensive drug-resistant TB [25].

The prevalence of antimicrobial resistance in Vietnam is among the highest in Asia, with 71% penicillin resistance and 92% erythromycin resistance for *Streptococcus pneumoniae*, 25% carbapenem resistance for *Pseudomonas aeruginosa*, and 40% for *Acinetobacter baumannii* [26].

### 3.2. Survey Results

#### Demography and Resource Allocation

Table 1 presents the demography, critical care resources, and POCT in all 15 hospitals surveyed (Levels 2, 3, and 4). The number of physicians on average ranged from 1 (SD, 0.49) in CHCs to 27 (SD, 9.9) in DHs to 248 physicians available in the PH. Vietnamese ambulances transported patients between hospital levels rather than picking patients up from home. Community health centers did not have ambulances on-site and were required to call 115, an emergency medical service call center number to request an ambulance to transport critically ill patients from the CHC to a referral site. Both DHs and the PH had two ambulances on-site to transfer critically ill patients.

Community health centers lacked adequate microbiology laboratories, critical care resources, hematology, and hemostasis testing. District hospitals were unable to measure arterial blood gas. Other critical care resources were limited with an average of 3 (SD, 2.7) pulse oximetry instruments and measuring electrolytes (43%). One CHC had two diagnostic tests to detect malaria (14%, 1/7), while the remaining CHCs (86%, 6/7) had no diagnostic tests available. District hospitals had microbiology laboratories but few diagnostic tests available to detect malaria (43%, 3/7), dengue fever (29%, 2/7), rubella (14%, 1/7), hepatitis A (29%, 2/7), hepatitis C (43%, 3/7), blood culture (29%, 2/7), and pathogen culture (57%, 4/7). The PH had broader critical care resources and microbiology tests (e.g., H1N1 or H7H9). Both Level 2 and Level 3 hospitals had HIV tests; however, none were found at Level 4 hospitals. Point-of-care testing was often scarce in CHCs and undersupplied in DHs, but the PH offered more tests per department.

Table 2 outlines the survey results for diagnostic tests and POC tests used by physicians to diagnose patients. Community health centers relied heavily on the syndromic management of patients with infectious diseases, HIV/STDs, sepsis, digestive/respiratory symptoms, and asthma. The PH had rapid diagnostic tests for infectious diseases; however, tests were performed in a clinical laboratory and not near patients’ bedsides. The PH rarely transferred patients to the Level 1 hospital and only transferred in severe cases. District hospitals lacked adequate diagnostic tests and treatment for patients with sepsis. One DH reported 66% of patients with sepsis died due to prolonged time taken to receive blood culture results. 

Table 3 summarizes medical problems in Central Vietnam hospitals and communities. Diabetes and hypertension were among the most neglected public health challenges which affected the working class, and the lack of disease prevention was highlighted as a public health and medical problem by one CHC and DH. Patients commonly infected with HIV, TB, sepsis, malaria, and dengue fever could not be treated at many CHCs. A few DHs could not treat patients with sepsis or dengue fever. The PH could not treat patients with either TB or HIV.

Table 4 focuses on acute medical response in the cases of healthcare emergencies or disasters by hospital levels. Level 4 hospitals were equally likely to seek emergency assistance as Level 1, 2, and 3 hospitals. No helicopter rescue was available.

Table 5 reports therapeutic turnaround times (TTAT) for tests sent to referral laboratories. The Level 2 hospital sent L-lactate tests to the Level 1 hospital and results were received back within 7 to 24 h. Level 3 hospitals sent patients’ tests for HIV, TB, dengue fever, malaria, and chlamydia to either the Center for Disease Control (CDC), the Center for HIV/AIDS Control, the TB and Respiratory Disease Center, HUH, or HCH. Test results were received ranging anywhere from 7 h to 30 days. Level 4 hospitals in general did not have diagnostic testing capabilities and were required to transfer the patient by calling 115.

**Figure 2:**
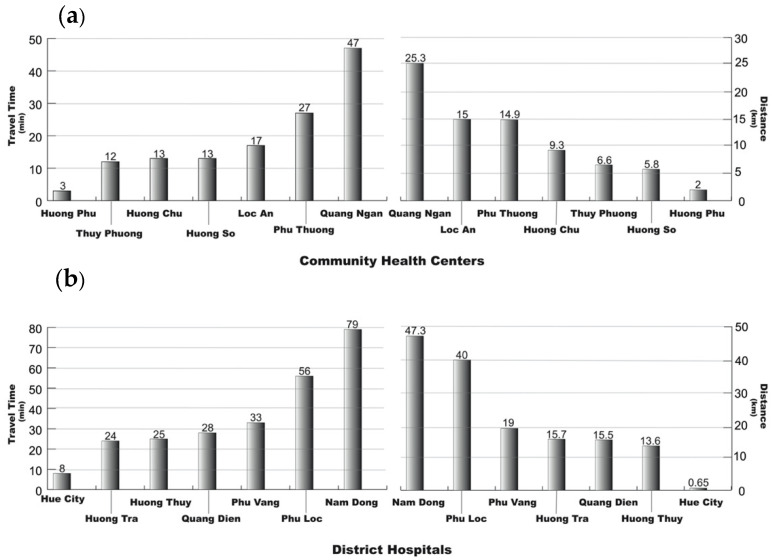
Travel time (min) and distance (km) from community health centers (**top**) and district hospitals (**bottom**) to their referral sites. (**a**) The top pareto plot of transport time (**left**) from the community health centers to either nearest district hospital, Hue University Hospital, or Hue Central Hospital. The corresponding distances for community health centers are shown as well (**right**). In rural regions such as Quang Ngan it took nearly fifty minutes for a patient to travel from the community health center to reach the referral site, whereas, in urban areas, travel time from Huong Phu community health center was less than five minutes to reach the referral site. (**b**) The bottom pareto plot shows the transport time (**left**) from the district hospitals to Hue University Hospital or Hue Central Hospital. The corresponding distances (**right**) for the district hospitals are shown as well. More mountainous regions such as Nam Dong took almost eighty minutes for a patient to travel from the district hospital to reach the referral site, whereas, in Hue City, patients traveled from their district hospital in less than ten minutes to reach the referral site.

The top graph in Figure 2a displays the distances and travel times between the surveyed CHCs and their referral hospitals. On average, it took 18.86 min (SD, 14.31) to reach the referral site at an average distance of 11.27 km (SD, 7.8) from the surveyed CHCs. The bottom graph in Figure 2b shows the distances and travel times between the surveyed DHs and their referral hospitals. On average, it took 36.14 min (SD, 23.70) to reach the referral site at an average distance of 21.68 km (SD, 16.23) from a DH.

Table 6 presents the site-specific placement of POC resources in primary care centers, patients’ homes, and emergency medical services. The Level 2 hospital and the Level 4 hospitals suggested complete blood cell count analyzers, blood gases, electrolytes, and blood typing be available at primary care centers. Level 3 hospitals suggested complete blood cell count analyzers, urinalysis, and tests to detect HPV, malaria, hepatitis B, HIV, and *H. pylori* should be available at primary care centers. In two instances, Level 4 hospitals suggested the placement of HIV tests in primary care centers. Level 2, 3, and 4 hospitals proposed glucose meters be placed in patient homes. In one instance, a Level 3 hospital recommended POC complete blood cell count and electrolyte analyzers be placed in patient homes. Level 2, 3, and 4 hospitals suggested POC blood gas analyzers be placed with emergency medical services (EMS). Level 2 and Level 4 hospitals recommended placing complete blood cell count analyzers with EMS and, in one instance, a Level 4 hospital suggested placing tests for malaria with EMS.

Table 7 reports hospital preparedness for highly infectious diseases. All hospital levels were unprepared for highly infectious disease outbreaks. All levels reported no isolation facilities near or outside the main hospital; however, each level reported staff trained to use personal protective equipment (PPE). The Level 2 hospital had an isolation laboratory within the main hospital and was capable of detecting TB via a PCR instrument. Table 8 presents hospital preparedness for patients with Ebola virus disease (EVD). All levels lacked adequate diagnostic tests to detect EVD and were unprepared for patients suspected of having EVD. All hospital levels lacked proper isolation facilities and directly transferred EVD patients to referral sites by ambulance.

Table 9 presents government directives and test clusters which can be used to diagnose patients with infectious diseases. All hospital levels were unable to detect patients with Zika virus. Level 1 and 2 hospitals had RT-PCR tests available for the identification of SARS-CoV-2. Level 1, 2, and 3 hospitals were capable of detecting microcephaly late in the second trimester or early in the third trimester of pregnancy by using an ultrasound. Level 4 hospitals were unable to detect dengue fever, syphilis, cholera, TB, HIV, or methicillin-resistant staphylococcus aureus (MRSA). No government directives were available for MRSA. No specific tests were mentioned in the government directives for severe acute respiratory syndrome (SARS). Molecular diagnostics such as GeneXpert MTB/RIF, capable of detecting resistance TB, were only available at the TB and Respiratory Disease Center. All hospital levels were unable to detect EVD and Middle East respiratory syndrome coronavirus (MERS-CoV). Diagnostic instrumentation such as RT-PCR and PCR were only available at the Level 1 and Level 2 hospitals; however, both lacked the necessary PCR reagents to detect Zika, EVD, MERS-CoV, and cholera. According to an official from the Microbiology Department at Hue University Hospital, reagents for cholera take 2–4 weeks to arrive. Tests to confirm Zika virus, EVD, and MERS-CoV must be sent to facilities authorized by the Ministry of Health, such as the National Hospital of Tropical Disease, National Institute of Hygiene and Epidemiology, or Pasteur Institute in Ho Chi Minh City (HCMC).

Table 10 reflects hospital levels’ diagnostic test availability and methods used for emerging pandemic and epidemic threats, bloodborne pathogens, foodborne illnesses, and vector-borne diseases. Due to the pandemic, there was a ubiquitous supply of COVID-19 rapid antigen tests (RAgTs) at all surveyed Level 2, 3, and 4 hospitals. The PH and one DH reported the capability of running PCR tests for COVID-19. In general, Level 4 hospitals lacked diagnostic testing for emerging pandemic and epidemic threats, bloodborne pathogens, foodborne illnesses, and re-emerging infectious diseases. One Level 4 hospital had a rapid diagnostic test (RDT) and Giemsa stain for malaria; however, no diagnostic tests for dengue fever were available. Level 3 hospitals had few diagnostic tests, but all had RDTs for HIV and hepatitis B. The PH had broader diagnostic testing capabilities, but all health facilities lacked public health preparedness for highly infectious disease threats (Ebola, MERS-CoV, SARS, and Zika).

## 4. Discussion

### 4.1. Geographic Optimization

On-site diagnostic testing should be integrated into public health strategies for isolation and disease control. Point-of-care instruments distributed along a SWN will enhance crisis preparedness and public health resilience. Disease surveillance capacity can be strengthened by trained nonlaboratory staff capable of testing and counseling patients in resource-limited settings such as CHCs and DHs deficient in health professionals [27]. Diagnostic tests made available upstream near patients’ homes in the SCP^TM^ can prevent the burden of overwhelming diagnostic delays and overcrowding downstream [28]. Collated epidemiological data from historical outbreaks have indicated that diagnostic delays contribute to epidemic size [29]. The risks associated with the emigration of humans and pathogens across geographical and political boundaries can lead to the rapid spread of disease into developing pandemics.

**Figure 3:**
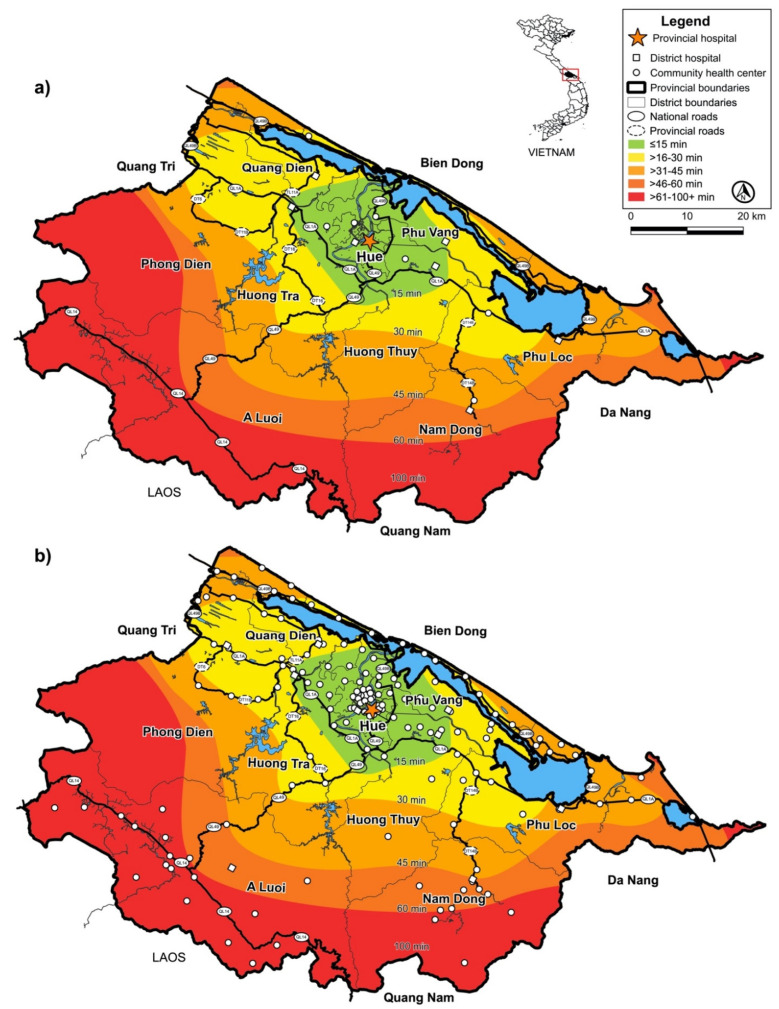
(**a**) **SWN time contours of surveyed health facilities.** Thua Thien Hue Province SWN time contours (**top**) illustrate the temporal and geospatial domains for the community health centers and district hospitals to reach Hue University Hospital. Physical road maps are shown by highways illustrating the most efficient routes patients take by traveling from one site to a referral site downstream of the SCP. Travel times (colored contours) and main roads (bold black) are shown. Surveyed community health centers in the red zone should perform rapid POCT on-site to allow patient transport directly downstream and bypass the district hospital while reducing TTAT for rapid treatment and isolation. All community health centers surveyed lacked microbiology facilities. Thus, POC pathogen detection would be useful in all zones. (**b**) **District hospitals and community health centers in Thua Thien Hue Province**. The time contours map (**bottom**) illustrates the temporal and geospatial domains between all community health centers and all district hospitals to reach Hue University Hospital. Influx of travelers from Laos, Quang Nam, Da Nang, and Quang Tri requires several hours, as they will be required to travel in a stepwise fashion through each level before reaching interventional care. Community preparedness can be enhanced with POCT in community health centers in the red zone that lack adequate diagnostic testing. POCT available near patients in community health centers and district hospitals could serve as a hub for incoming travelers and locals who require rapid medical response.

In Figure 3a (top), in mountainous regions such as Nam Dong, it is shown that it took nearly eighty minutes for a patient to travel from the DH to reach the referral site, whereas, in Hue city, patients traveled from their DH in less than ten minutes to reach the referral site. In rural regions such as Quang Ngan, it took nearly fifty minutes for a patient to travel from the CHC to reach the referral site, whereas, in urban areas, a patient’s travel time from Huong Phu CHC was less than five minutes to reach the referral site. The SWN time contours illustrate the temporal and geospatial domains for the CHCs and DHs to reach HUH. Physical road maps are shown by highways illustrating the most efficient routes patients take by traveling from one site to a referral site downstream of the SCP. Surveyed CHCs in the red zone should perform rapid POCT on-site, which can allow patient transport directly downstream by bypassing the DH while reducing TTAT for rapid treatment and isolation. The field deployment of rapid diagnostic tests in geographically isolated areas could aid in identifying patients with a highly infectious disease in remote communities. All CHCs (Level 4 hospitals) surveyed lacked microbiology facilities. Thus, POC pathogen detection would be useful in all zones.

In Figure 3b (bottom), TTHP SWN time contours illustrate the temporal and geospatial domains for all CHCs and DHs in TTHP to reach HUH. Many CHCs in the red zone lacked adequate treatment and isolation. Incoming travelers from Quang Nam, Da Nang, and Quang Tri would be required to travel in a stepwise fashion through each level before reaching interventional treatment. POCT implemented near patients in CHCs and DHs can help achieve timely evidence-based decision making based on the POC test results for incoming travelers and locals who require rapid medical response.

### 4.2. Disease Surveillance Capacity

A key challenge in infectious disease (e.g., COVID-19, Zika, malaria, EVD, dengue virus, etc.) surveillance is the detection and geographic distribution of patients with the disease [30]. Infectious diseases spread in patterns characteristic of SWNs. Infected patients can spread the disease to others as soon as symptoms appear, as with EVD and even before symptoms for COVID-19, requiring quick identification and isolation to mitigate disease outbreaks. Public health surveillance utilizing the contact tracing of suspected infected individuals can be useful for containing highly infectious threats, especially when POC test results are known early on [31,32,33,34].

**Figure 4:**
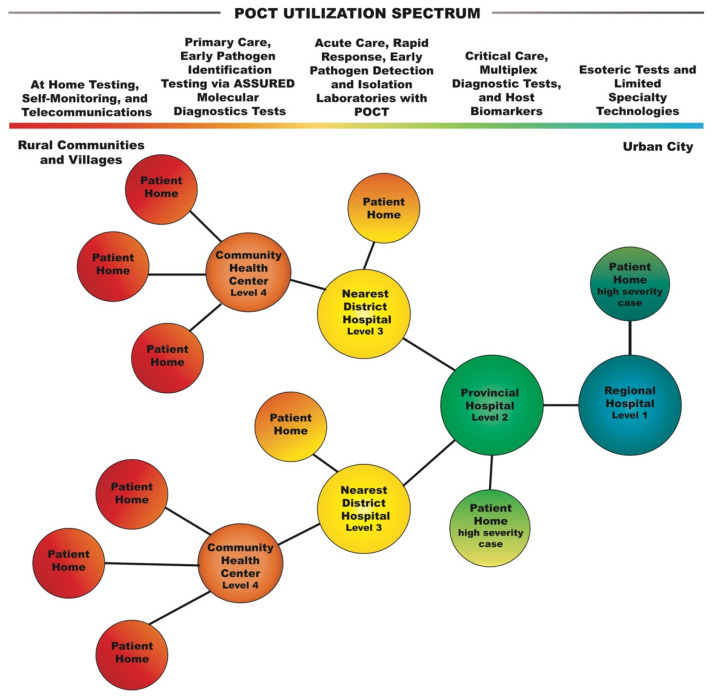
**Healthcare network and point-of-care testing spectrum**. Limited resource settings in rural communities and villages can take advantage of the POCT spectrum for accessibility to diagnostic testing near their homes, which maximizes healthcare efficiency. The current healthcare network requires patients in rural communities to travel over an hour for diagnosis and interventional care.

A person with EVD can transmit the disease once symptomatic [30]. Many hospitals in developing countries have embedded POCT to manage EVD transmission by providing timely and accurate diagnostics [12,19,34]. Hence, detection at the initial points of patient contact minimizes spread and prevents outbreaks from occurring [35]. The geographically optimized placement of POC tests near patients’ homes can prevent high-risk transmission from traveling downstream in the SCP (see Figure 4). Community health centers and DHs are limited to glucose tests, pregnancy tests, urine tests, and electrocardiographs (ECGs). Basic critical care companion tests (electrolytes, blood gases, etc.) can be integrated in CHCs and DHs upstream to serve more patients near their homes and reduce the risk of transmission from occurring downstream in the SWN.

Spatial Care Paths^TM^ can expedite patient care from initial points of contact with diagnostic tests to interventional care and isolation by eliminating time-consuming steps and risks associated with transportation from one hospital level to the next. All CHCs and many DHs lacked proper diagnostic and laboratory equipment, requiring patients to move downstream in the SCP. Portable POC diagnostic tests are effective in screening patients in remote villages and communities with high patient volumes per physician. Emergency medical service staff with on-hand diagnostic tests can assess a patient’s status and contribute to the decision to transfer a patient to a health facility with an isolation unit. Point-of-care tests can deliver value to clinical decision making for patients suspected of having sepsis, where delay in appropriate treatment can be fatal.

Vietnamese rural health is affected by a lack of accessibility to adequate healthcare close to home, especially when resources are concentrated in the city. Point-of-care testing utilization can help dissolve healthcare inequity between patients in rural and urban regions (see Figure 4). In rural limited resource settings, rapid POC tests can help drive patients to seek medical attention in a timely manner before symptoms become severe.

### 4.3. Essential Diagnostic Tests, Antimicrobial Stewardship, and Isolation Laboratories

The WHO Essential Diagnostic List is a useful start for Vietnamese policy-makers to adopt tests to implement at the national level [36]. The limited availability of POCT can overwhelm the central laboratory. Point-of-care testing can fulfill diagnostic testing demands, accelerate management, extend laboratory testing within hospital departments, and reduce TTATs and patient readmissions [28]. Multiplex diagnostic tests can be beneficial when pathogens create similar clinical symptoms and signs, as in the case of EVD, or when samples are difficult to collect or short on volume [31,32,33,34].

Evidence-based devices, such as an I-Stat^®^ device (Abbott, Abbott Park, Illinois, United States) made accessible at the patient bedside, proved reliable in an Ebola treatment center, in ICUs, and in the aftermath of the Haiti earthquake of 2010 for monitoring blood gas analysis, coagulation, and basic biochemistry testing [37]. A study by Strecker et al. [38] found a capillary blood specimen could serve as an alternative to a venous blood sample for the rapid diagnosis of Ebola virus infections in resource-limited settings. Similarly, the REEBOV rapid diagnostic test kit has high sensitivity and specificity at the POC in rural and laboratory settings, allowing healthcare workers to quarantine, monitor, and provide supportive treatment (e.g., rehydration, electrolytes, antibiotics, and antimalarials) to the patient [39,40]. Similarly, the TB-LAM (Alere, Waltham, MA, USA) is a rapid diagnostic test, capable of detecting TB within 25 min and costing less than USD 3 per test [41]. The matrix-assisted laser desorption/ionization time-of-flight mass spectrometer (MALDI-TOF MS) is a fast, accurate, and inexpensive identification system compared to other immunological or biochemical tests and is predicted to become the standard method for pathogen identification in the microbiology laboratory [42]. A study by Chan et al. [43] used a thermos thermal cycler to detect STDs, EVD, HIV/AIDS, and dengue virus, costing less than USD 200 to build and able to test eight specimens at a time.

#### 4.3.1. Avian and Seasonal Influenza Tests

Rapid influenza diagnostic tests (RIDT) can enable physicians in Vietnam to institute prompt measures of infection control during seasonal influenza outbreaks in remote settings or when the central laboratory is overwhelmed [44,45]. The current risk posed by the Asian-lineage H7N9 influenza (pandemic influenza) virus was rated by the Influenza Risk Assessment Tool as having the greatest potential to cause a pandemic, as well as potentially posing the greatest risk to public health [46].

According to the Clinical Laboratory Improvement Amendments of 1988 (CLIA), cleared RIDTs can produce results within 15 min at the POC and can help identify seasonal influenza and help minimize prescribing unnecessary antibiotics. Real-time RT-PCR molecular assays are the preferred method for identifying the H7N9 influenza virus [45,47].

#### 4.3.2. Antimicrobial Resistance Susceptibility Testing

The early detection of MRSA on admissions or in intensive care units where patient vulnerability is great can reduce spread and the risk of the development of sepsis. In a suspected septic patient, blood cultures can be costly, especially if targeted antimicrobial treatment is not administered within six hours. Point-of-care molecular diagnostics can help physicians identify a causal pathogen and provide targeted antimicrobial treatment. However, AMR is a major factor which results in clinical unresponsiveness to treatment and progression to septic shock [48]. The accurate diagnosis of septic patients can mitigate unnecessary antibiotic use [49]. The lack of proper monitoring of vital signs, which is unavailable in Level 4 hospitals, is necessary to guide the clinical management of patients with sepsis. Antimicrobial susceptibility testing is performed in Level 1, 2, and 3 hospitals. However, a majority of antibiotics being prescribed are in primary care settings by Level 4 physicians, where no antimicrobial susceptibility testing can be performed. 

Infected individuals with EVD have overlapping symptoms with malaria and cholera (electrolyte imbalances), which makes it difficult to diagnose, as patients are simply treated on the basis of a constellation of nonspecific symptoms and signs that are clinically indistinguishable from other coendemic diseases [50,51,52]. Antibiotics are a cornerstone for the management of bacterial infections, which should be used with caution and only when necessary. Rapid POCT in microbiology laboratories in conjunction with antimicrobial guidelines and policies, support from antimicrobial pharmacists, heterogenous antibiotic use, and a reduction in the length of antimicrobial chemotherapy can help deliver antimicrobial stewardship [42].

#### 4.3.3. COVID-19, Zika, and Malaria Tests

The COVID-19 pandemic, now in its third year, has confirmed the need for point-of-care testing at points of need. Currently, RT-PCR and RAgTs are available at the Level 1 and 2 hospitals. Level 3 hospitals in and around Ho Chi Minh City (HCMC) have been retrofitted to serve as isolation and treatment wards for those suspected to have or infected with COVID-19 [53]. Many COVID-19 RAgTs are now ubiquitous and accessible for people suspected of having COVID-19 in pharmacies throughout TTHP and at Level 3 and 4 hospitals by appointment.

In diagnosing patients with Zika virus, Level 1, 2, and 3 hospitals in Central Vietnam primarily rely on fetal ultrasound as their diagnostic tool. However, according to the CDC, there is no optimal timing or accuracy to detect microcephaly using fetal ultrasound among pregnant women with possible maternal Zika virus exposure [54]. The accuracy of microcephaly detection is influenced by many factors, such as the time of maternal infection relative to the time of screening, severity, patient factors (e.g., obesity), gestational age, equipment used, and the expertise of the person performing the ultrasound [54,55]. The FDA has issued Emergency Use Authorization (EUA) for several molecular diagnostic tools for detecting the presence of Zika virus [30,54,55].

Since 1958, the testing and diagnosis of malaria has been available free of charge for the Vietnamese public sector. However, few malaria diagnostic tests were available across surveyed primary care centers and DHs in Central Vietnam [56].

#### 4.3.4. Sexually Transmitted Disease Tests

A strategy for early intervention or treatment is screening [34]. In CHCs, the immediate treatment of curable sexually transmitted diseases (STDs) has often been based on syndromic management. Symptom management is limited in identifying asymptomatic infections (false negatives) and leads to overtreating patients (false positives) without proper laboratory testing. Community health centers and DHs lack adequate STD testing (see Table 10). Improving testing coverage and reducing time to treatment by implementing rapid POC tests for chlamydia, gonorrhea, syphilis, herpes simplex virus (HSV), human papillomavirus (HPV), and HIV in Central Vietnam can help physicians avoid unnecessary treatment and the risk of antibiotic resistance. A study by Guy et al. [57] showed that routine testing using the GeneXpert platform in Australia’s remote primary care settings reduced the prevalence rate of STDs [58]. Simple multiplex POC tests for syphilis and HIV are relatively simple and can be used outside reference laboratory infrastructures in CHCs and patient homes [59]. Accessible POC HIV, chlamydia, and gonorrhea testing proved essential to early diagnosis, the initiation of targeted treatment, and increased life expectancy in remote primary care settings [58,60]. Hence, the decentralization of multiplex POC tests for STDs in primary care settings offers a practical solution for screening [61].

#### 4.3.5. Testing for Hepatitis 

Hepatitis viruses (HBV and HCV) have a high prevalence in Vietnam, with approximately 8.6 million infected individuals [62]. Remote primary care settings capable of HBV testing and vaccinations in real time can alleviate disease burden. In a study by Smith et al. [63], rural villages in Guatemala with access to POC HBV testing improved linkage to clinical care and made HBV-negative individuals eligible for vaccinations. Point-of-care hepatitis C tests are inexpensive and do not require laboratory infrastructure or skills. Both HBV and HCV are generally asymptomatic and prone to misdiagnosis and result in delayed care for symptomatic individuals.

#### 4.3.6. Isolation Laboratories

Level 3 and 4 hospitals lack isolation facilities. Patients who display Ebola hemorrhagic fever are immediately isolated without the use of central laboratories, which may cause contamination and health facilities to shut down similarly to countless COVID-19 outbreaks that left hospitals inoperable [64]. Community isolation centers equipped with POC tests can provide early detection and disease surveillance to prevent spread in local and regional SWNs. The construction of isolated laboratories with POC molecular diagnostic tests near the patient can fulfill the regulatory and safety requirements of a BLS4 facility [35]. Isolation beds can house suspected patients and separate them from other patients in order to avoid the transmission of the disease. In the absence of proper isolation, patients can transmit viruses and resistant bacterial organisms to healthcare staff, the elderly, children, and pregnant women.

### 4.4. Diagnostic Test Criteria and Placement

According to WHO, the least number of steps to complete a diagnostic test is ideal: minimal preparation steps (required), <3 steps (desired), and <10 steps (acceptable) [32,33]. An ideal diagnostic test should follow the ASSURED principles: A—affordable, S—specific, S—sensitive, U—user-friendly, R—rapid and robust, E—equipment-free, and D—deliverable to stakeholders [50,65]. If more than one POC test is available, Vietnamese public health professionals can make a diagnostic test selection based on performance, environmental specifications, portability, sensitivity, specificity, and predictive values to prepare for disease outbreaks. 

POC test placements in different-level hospitals and on ambulances should be optimized based on SCP designs for rescuing, diagnosing, and treating patients rapidly [34]. A patient who perceives themselves as critically ill can directly visit HUH or HCH. However, many patients remain uncertain of the severity of their illness and may first travel to a CHC and then a DH before being transported to either a HUH or HCH. Geospatial analysis can utilize limited-resource implementation strategies for the placement of health resources to be used in disasters and disease outbreaks that may impact the healthcare system [66,67]. Thus, GIS can be used to streamline clinical decision making upstream in the SCP in limited-resource settings, which can benefit from accessibility to diagnostic testing near patient homes (see Figure 4).

Point-of-care testing programs can be designed and implemented in rural and remote areas to educate and train healthcare staff on the use of RAgTs for infectious diseases [68]. Test clusters can be selected for types of disasters [69,70]. Brock et al. [71] recommended the following pandemic diagnostic tests health facilities: Influenza A/B, H1N1, avian H5N1, EVD, SARS, MERS-CoV, Zika, and MRSA for bloodstream pathogens. It makes perfect sense to use affordable POC tests which require very little infrastructure and take less time in triaging patients without contaminating central laboratory services, meanwhile, being highly sensitive and specific [11].

### 4.5. Limitations

There are several limitations to this study. We surveyed a small sample of CHCs and DHs in TTHP, which limited our ability to characterize data distributions, make quantitative statistical comparisons, and generalize. Our results could have been better represented if all CHCs and DHs were surveyed in TTHP. Due to language barriers, the accuracy of translations could have been misconstrued even with the assistance of medical student translators. Due to the COVID-19 pandemic, data collection on COVID-19 was derived through MoH publications, email discussions, and dialogues but may not reflect the true impact it has had on patients across the healthcare system in Central Vietnam.

## 5. Conclusions

- ***Needs assessment***. The hospitals surveyed in Central Vietnam lacked adequate resources and preparedness for the rapid diagnosis of highly infectious pathogens at both point-of-care and in-hospital laboratories. A lack of POC infectious disease testing in the rural communities and distant geographical regions of TTHP meant that patient access and routes to rapid interventional care and isolation for highly infectious diseases was compromised. Evidence-based decision making was deficient in primary care centers. In order to improve healthcare access, improving availability and accessibility to rapid diagnostic information can be a catalyst in optimizing a public health framework by making it more resilient for local communities.
- ***POC coordinators***. POC coordinators are essential in implementing POCT, assuring the quality control of instrumentation, and are valuable in facilitating accreditation (e.g., ISO 22870) [72]. POC coordinators should be able to oversee several primary care centers on a daily basis to ensure the proper use of test results.
- ***Novel technologies***. Novel technology (paper-based or smartphone-based) with enhanced accuracy, portability, and response can reduce the TTAT to care and costs in decentralized health settings [73]. Multiplex molecular diagnostics for infectious diseases utilize large test clusters to screen for various pathogens and avoid substantial delays in TTAT to treatment and improve patient health outcomes [74].
- ***Upstream shift *via* SCP^TM^***. Point-of-care testing placed upstream in SCPs^TM^ (see Figure 3) can reduce timely critical decision making for patients presenting with disease-like symptoms of highly infectious diseases in the community. Point-of-care testing placement upstream offers a unique ability to detect infected patients that must be quarantined and isolated.
- ***Geographic information system***. The incorporation of GIS and temporal analysis can play an important role in visualizing inefficiencies in current healthcare access and understanding how patients utilize health networks. Geospatial analysis can help visualize the redistribution of health resources and the placement of POC tests to be used in case of disasters (e.g., tsunamis or earthquakes) and disease outbreaks that may impact healthcare systems, especially rural healthcare.
- ***Isolation laboratories/units***. The COVID-19 pandemic, EVD, and MERS-CoV crises proved unequivocally the importance of POCT usage in isolation laboratories. Due to the high risk of contamination and inoculation, if patients’ specimens sent to the clinical laboratory are aerosolized, spilled, or broken, it would shut down centralized laboratory testing and cripple hospital functions. POC testing with integrated diagnostic test clusters delivered in compact packages to an isolation laboratory/unit would shorten test processing and help eliminate unnecessary centrifugation steps.

### Future Recommendations

**Figure 5:**
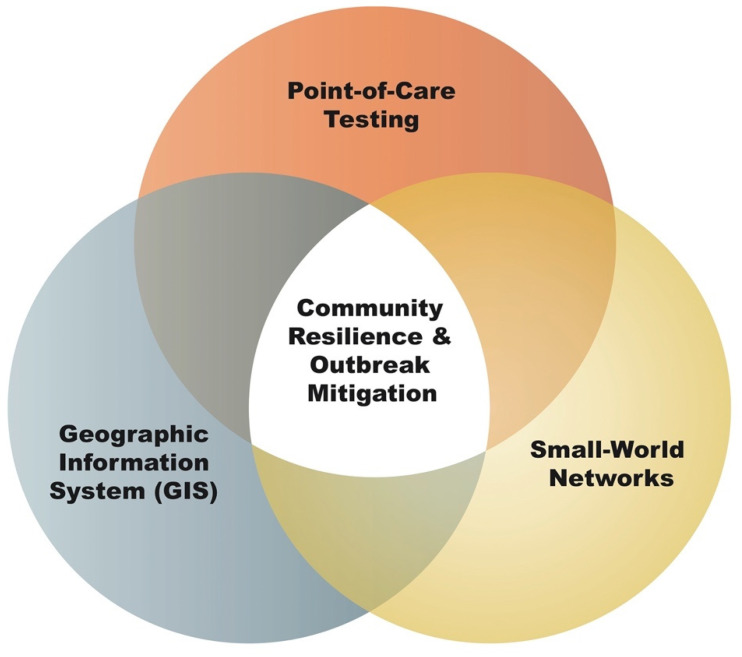
**Dynamic model to enhance community diagnostic capabilities**. Community resilience can be enhanced through the dynamic relationship between GIS, SWN, and POCT. GIS plays an important role in identifying geospatial relationships between health resources in SWNs. By optimally incorporating POCT in SWN, vulnerable health resource facilities may obtain diagnostic medical information that drives clinical decision making upstream of SCP.

- ***Community resilience***. Community resilience can be enhanced through a dynamic relationship between GIS, SWN, and POCT. We recommend that actionable evidence gathering in a SWN should shift to individuals in homes, primary care centers, and generally upstream in the SCP where it is needed the most, for the early detection of highly infectious diseases. Point-of-care tests near patients’ homes for immediate diagnosis at points of need can enhance public health readiness and resilience by minimizing the effects of outbreaks in remote areas (see Figure 5).
- ***Public health preparedness.*** Health institutions and practitioners in Vietnam can work with the Provincial People’s Committee and Commune People’s Committee to decrease the likelihood of disease transmission by ensuring all personnel from all levels (Levels 1, 2, 3, and 4) can provide rapid diagnosis and treatment and reduce the risk of transmission or reinfection. Additional supply lines for rapid multiplex molecular diagnostics tests need to have adequate stock in neighboring areas that can be affected by ill-prepared travelers.
- ***Point-of-care testing education***. Public health practitioners and nonlaboratory staff should participate in the training and assessment of how to operate POC tests in simulated scenarios [75,76].
- ***National POCT and antimicrobial stewardship policies and guidelines***. We recommend Vietnam invest in public health preparedness by establishing health technology assessments for POC tests along with national POCT policies and guidelines so new technologies can meet public health needs, enhance laboratory capacity in rural regions, and provide early detection. Point-of-care tests can be properly adapted to cultural expectations and be cost-effective. Healthcare policies should reflect solutions to increase public health preparedness, surveillance, prevention, and treatment. Vietnam’s leadership could help harmonize the standard of practice of POC testing for infectious diseases throughout the ASEAN (Association of Southeast Asian Nations) region. Multiplex POC tests capable of detecting resistant organisms can provide accurate diagnosis and treatment options based on drug susceptibility testing [49,77]. The rampant usage of antibiotics prescribed and sold in TTHP can lead to the emergence of antimicrobial-resistant pathogens. Multiplex POC tests can be implemented in AMR national and regional action plans that meet the *Asia Pacific Strategy for Emerging Diseases and Public Health Emergencies* (ASPED III) strategic goals [1].
- ***Geographic optimization***. An analysis of travel times between hospitals, ambulance routes, and telecommunication systems can reveal the optimal rescue and interventional sites for the POC infectious disease testing placement, along with simultaneous consideration of routing patients from homes and primary care sites to upstream hospitals where isolation and quarantining may be available.
- ***Point of care culture.*** Providing culturally sensitive and user-friendly POC diagnostic services in community settings can increase healthcare workers’ and patients’ trust [78]. Training and diagnostic procedures should be translated into local and ethnic languages (e.g., Cơ Tu or Tà Ôi) and adapted to community needs.
- ***Emergency medical services (EMS)***. We recommend EMS services be equipped with POC tests in the SWN, especially near rural communities and environmental regions far from definitive care. SCPs should be designed so that the TTAT is less than one hour for improving patient outcomes. Mobile rescue with on-hand POCT can accelerate diagnosis upstream with telehealth connectivity.
- ***Developing a task force for studying and making decisions about point-of-care tests***. The goal of a POC task force is to make cost-effective recommendations regarding the use of and reimbursement of POC tests for at-risk patient populations. A POC task force weighs the benefits for POCT implementation and regulatory barriers related to the licensing of medical laboratories. A task force can urge the Vietnamese Ministry of Health to consider policy and guidelines in order to have a sound basis for implementing new POC technologies that align with stakeholders. Any decision to promote the widespread use of POC testing should be accompanied by outreach to medical providers to actively utilize POCT to eradicate disease burdens.

## Figures and Tables

**Table 1:** Demography, critical care resources, and point-of-care testing utilization in Level 4 (7), Level 3 (7), and Level 2 (1) hospitals in Central Vietnam.

	Level 4 Hospitals	Level 3 Hospitals	Level 2 Hospital
	N = 7	N = 7	N = 1
**Demography** **(Mean (SD))**			
No. of daily patient visits	33 (18.4)	256 (206.5)	410
No. of beds	7 (2.6)	128 (65.9)	605
Physicians	1 (0.49)	27 (9.9)	248
Nurses	1 (0.38)	40 (19.9)	150
Pharmacists	1 (0.49)	9 (5.5)	17
No. of ambulances ^a^	0	2 (0)	2
Diagnostic laboratory service			
Weekdays	1 site ^c^; 7 h 45 min	8 h (0.35)	8 h
On-call service provided	1 site ^b^	6 sites ^b^	Available
POCT program	No	No	No
POCT director	No	No	No
POC coordinator	No	No	No
**Critical Care Resources**			
No. of SpO_2_ monitors (mean (SD))	0	3 (2.7)	9
Blood gases	Not available	Not available	P_O2_, P_CO2_, pH, and HCO_3_^−^
Electrolytes	Not available	3 sites ^b^; Na, Cl, K, and Ca^2+^	Na, Cl, K, and Ca^2+^
Microbiology (frequency)			
	Laboratories (0)	Laboratory (100)	Laboratory
	Giemsa stain (14)	HCV-Ab (43) and pathogen culture ^c^ (57)	Pathogen culture ^c^
	Malaria Ag (14)	Dengue fever (NS1, IgG, and IgM) (29)	Blood culture, HIV, and HEV
		Malaria Ag (43) and HBsAg (100)	Influenza A, B, H1N1, and EV71
		Pap smear (14), HAV-Ab (29),	H7H9, strep throat, HAV-Ab, and
		rubella test (14), and HIV test (100)	dengue fever (NS1, IgG, and IgM)
		HBsAb (29) and blood culture (29)	Rubella test
	Dark field microscopy for syphilis (14)	Gram-stained smear for TB	
		Gonorrhea gram-stained smear (14)	HBsAg, HBeAg, and HCV-Ab
		H. pylori-Ab (14)	Pap smear, malaria Ag, fecal smear, Toxoplasma-Ab, CMV-Ab, PCR, dark field microscopy, syphilis rapid test, H. pylori-Ab, Tzanck smear, chlamydia rapid test, and gram-stained smear for gonorrhea
Hematology	Not available	CBC	CBC
Hemostasis (frequency)	Not available	PT and aPTT (86)	PT and aPTT
		Bleeding time (14) and clotting time (14)	Bleeding and clotting time
**Point-of-Care Testing** **(Frequency)**			
Clinical wards	2 sites ^b^; urinalysis (57)	ECG (43), SpO_2_ (14), and BP (14)	Glucose meter and BP
	Ultrasound (14) and ECG (14)		SpO_2_ and ECG
	Glucose meter (14)		
ER	5 sites ^b^; malaria Ag (14)	ECG (57), SpO_2_ (71), and BP (43)	Glucose meter and SpO_2_
	BP (29) and glucose meter (14)	Glucose meter (29) and BT (14)	BP, ECG, bhCG, and cardiac monitor
	Ultrasound (29) and ECG (43)	bhCG (14)	
	bhCG (14)		
OR	No ORs available	ECG (43), SpO_2_ (71), and BP (43)	Glucose meter, SpO_2_, and EtCO_2_
		Glucose meter (29) and BT (14)	Cardiac monitor
		Ultrasound (14)	
ICU	No ICUs available	6 sites ^b^; ECG (29) and SpO_2_ ^d^ (57)	Glucose meter, SpO_2_, and EtCO_2_
		EMG (14) and ultrasound (14)	Cardiac monitor and ultrasound
		Glucose meter (14) and BP (29)	Central venous pressure and BP
CCU	No CCUs available	No CCUs available	No CCUs available
NICU	No NICUs available	6 sites ^b^; ECG (14) and SpO_2_ (29)	Echocardiograph
		EMG (14) and bilirubin (14)	
LR	5 sites ^b,e^; ECG (14) and CTG (29)	CTG (71), SpO_2_ (29), and EMG (14)	SpO_2_ ^f^, BP, and CTG
	BP (14) and ultrasound (14)	BP (29) and ECG (29)	Ultrasound

Abbreviations: aPTT, activated partial thromboplastin time; bhCG, beta-human chorionic gonadotropin; BP, blood pressure; BT, body temperature monitor; Ca^2+^, ionized calcium; CBC, complete blood cell count; CCU, coronary care unit; Cl, chloride; CMV-Ab, *cytomegalovirus antibody test*; CTG, cardiotocography; ECG, electrocardiograph; EMG, electromyography; EV71, enterovirus 71; ER, emergency room; EtCO_2_, end-tidal CO_2_; *E. coli*, *Escherichia coli*; H1N1, swine flu; H7N9, avian influenza A; HAV-Ab, hepatitis A virus antibody test; HBeAg, hepatitis B envelope antigen test; HBsAg, hepatitis B surface antigen test; HBsAb, hepatitis B antibody test; HCO_3_^−^, bicarbonate; HCV-Ab, hepatitis C virus antibody test; HEV, hepatitis E virus rapid antibody test; HIV, human immunodeficiency virus; H. pylori-Ab, helicobacter pylori antibody test; ICU, intensive care unit; IgG, immunoglobulin G; IgM, immunoglobulin M; K, potassium; LR, labor room; malaria Ag, malaria antigen; Na, sodium; NICU, neonatal intensive care unit; NS1, nonstructural protein 1; OR, operating room; PCR, polymerase chain reaction; P_O2_, partial pressure of oxygen; P_CO2_, partial pressure of carbon dioxide; pH, potential of hydrogen; PT, prothrombin time; SpO_2_, peripheral capillary oxygen saturation; TB, tuberculosis; Toxoplasma-Ab, toxoplasma gondii antibody test. Footnotes: ^a^ CHCs call 115 if they need to transfer a patient to upstream care. ^b^ The number of hospitals that responded. ^c^ Pathogen cultures of *E. coli*, *H. pylori*, dysentery, and salmonella were confirmed by both the provincial and district hospitals. ^d^ One wireless SpO_2_ monitor was being used. ^e^ Two out of five labor rooms encountered were allowed to perform deliveries, and the remaining three lacked staff and equipment. ^f^ Monitor using smartphone application.

**Table 2:** Diagnostic test utilization, POC test availability, and referral sites for patients with infectious diseases.

Survey Questions	Level 2 Provincial Hospital N = 1	Level 3 District Hospitals N = 7 (Response Frequency by Sites Surveyed)	Level 4 Community Health Centers N = 7 (Response Frequency by Sites Surveyed)
Diagnostic tests performed ^a^	HCV-Ab, EV71, HBsAg, HEV, malaria Ag, *H. pylori*, HIV, CBC, CRP, ultrasound, X-ray, urinalysis, and blood culture	CBC (5), X-ray (2), ultrasound (2), blood culture (1), and AFB (1)	None, rely on patient symptoms (7 ^f^); Giemsa stain (1); and malaria Ag (1)
POC tests available ^b,e^	SpO_2_, HCV-Ab, EV71, HBsAg, HEV, malaria Ag, *H. pylori*, and HIV	6 Sites ^d^; ultrasound (1), HBsAg (1), HCV-Ab (1), SpO_2_ (1), ECG (1), BP (1), and none (1)	None (4), ultrasound (3), ECG (2), urinalysis (2), malaria Ag (1), and BP (1)
Referral hospital ^c^	Hue Central Hospital in severe cases	Hue University Hospital (3); Hue Central Hospital (2); and none, treat at the district hospital (2)	Hue University Hospital (4), nearest district hospital (4), and Hue Central Hospital (3)
**Patients with HIV/Sexual Transmitted Diseases**			
Diagnostic tests performed ^a^	HIV rapid test, HBsAg, HCV-Ab, and pap smear	HIV rapid test (4); HBsAg (2); none, rely on patient symptoms (1); pap smear (1); gonorrhea gram-stained smear (1); CBC (1); urinalysis (1); and gram stain (1)	None, rely on patient symptoms (7)
POC tests available ^b,e^	HIV rapid test	HIV rapid test (4), HBsAg (1), and syphilis rapid test (1)	None (4) and urinalysis (2)
Referral hospital ^c^	Treat patient, do not transfer	Hue University Hospital (4) Hue Central Hospital (4), and Center for HIV/AIDS Control (2)	Hue University Hospital (4), Hue Central Hospital (3), nearest district hospital (3), and Center for HIV/AIDS Control (2)
**Patients with Sepsis**			
Diagnostic tests performed ^a^	CBC, urine creatinine, ALT, AST, electrolytes, procalcitonin, lactate, coagulation, and CRP	CBC (6); blood culture (2); ultrasound (1); and none, rely on patient symptoms (1)	None, rely on patient symptoms (7)
POC tests available ^b^	SpO_2_	SpO_2_ (2) and BP (2)	None (4), ultrasound (2), ECG (2), urinalysis (2), and BP (1)
Referral hospital ^c^	Treat patient, do not transfer	Hue University Hospital (6) and Hue Central Hospital (3)	Hue University Hospital (5), Hue Central Hospital (5), and nearest district hospital (2)
**Patients with Digestive Symptoms**			
Diagnostic tests performed ^a^	Ultrasound, CBC, CRP, amylase, and lipase	Ultrasound (6); CBC (2); X-ray (2); LFT (1); AST (1); ALT (1); stool smear (1); rectal, sigmoid, esophageal endoscopy (1); and none, rely on patient symptoms (1)	Ultrasound (4); none, rely on patient symptoms (4)
POC tests available ^b^	SpO_2_	6 sites ^d^; none (3), ultrasound (2), SpO_2_ (1), and BP (1)	Ultrasound (5), BP (1), and none (2)
Referral hospital ^c^	Treat patient, do not transfer	Hue University Hospital (5); Hue Central Hospital (3); and none, treat at the district hospital (1)	Nearest district hospital (4); Hue University Hospital (4); Hue Central Hospital (3); and none, treat at the community health center (1)
**Patients with Respiratory Symptoms**			
Diagnostic tests performed ^a^	CBC, blood gas, CRP, ECG, and PFT	X-ray (5); none, rely on patient symptoms (2); CBC (2); AFB (1); SpO_2_ (1); ECG (1); and fluorescent microscopy (1)	None, rely on patient symptoms (7)
POC tests available ^b^	SpO_2_ and ECG	None (4), SpO_2_ (3), ECG (2), integrated BP monitor (2), and cardiac monitor (1)	None (4), ultrasound (3), ECG (2), and BP (1)
Referral hospital ^c^	Treat patient, do not transfer	Hue University Hospital (5); Hue Central Hospital (3); and none, treat at the district hospital (1)	Hue University Hospital (4); Hue Central Hospital (3); nearest district hospital (3); none, treat at community health center (1); and Tuberculosis and Respiratory Diseases Center (1)
**Patients with Asthma**			
Diagnostic tests performed ^a^	CBC, blood gas, and PFT	CBC (3); none, rely on patient symptoms (2); SpO_2_ (2); AFB (1); X-ray (1); and PFT (1)	None, rely on patient symptoms (7)
POC tests available ^b^	SpO_2_	None (3), SpO_2_ (3), ECG (1), BP (1), and cardiac monitor (1)	None (4), ultrasound (3), ECG (2), and BP (1)
Referral hospital ^c^	Treat patient, do not transfer	Hue University Hospital (5); Hue Central Hospital (3); and none, treat at the district hospital (1)	Hue University Hospital (4), Hue Central Hospital (3), nearest district hospital (3), and Tuberculosis and Respiratory Diseases Center (1)

Abbreviations: AFB, acid-fast bacilli; ALT, alanine aminotransferase; AST, aspartate aminotransferase; BP, blood pressure; CBC, complete blood cell count; CRP, C-reactive protein test; ECG, electrocardiograph; EV71, enterovirus 71; HBsAg, hepatitis B surface antigen test; HCV-Ab, hepatitis C antibody test; HEV, hepatitis E virus rapid antibody test; HIV, human immunodeficiency virus rapid test; *H. pylori*, helicobacter pylori; LFT, liver function test; malaria Ag, malaria surface antigen test; PFT, pulmonary function test; SpO_2_, peripheral capillary oxygen saturation. Footnotes: ^a^ Survey question 3.29, ^b^ question 3.31, and ^c^ question 3.30. Respondents were allowed to give more than one answer per question. ^d^ Six district hospitals provided responses. ^e^ Many rapid POC tests for infectious diseases are not performed near patients’ bedsides. Patients’ blood is drawn and then taken to the laboratory 1–2 floors away for the actual test to be performed. ^f^ In most cases of infectious diseases, the patients’ clinical symptoms are primarily used.

**Table 3:** Medical problems in Central Vietnam hospitals and communities.

Survey Questions	Level 4 Community Health Centers N = 7 (Response Frequency by Sites Surveyed)	Level 3 District Hospitals N = 7 (Response Frequency by Sites Surveyed)	Level 2 Provincial Hospital N = 1
5.2. Which medical problems are neglected?	Diabetes mellitus (3), hypertension (3), hepatitis (A-E) (1), cancer (1), disease prevention (1), stroke (1), lipid profile (1), and none ^a^ (1)	Diabetes (4), hypertension (3), disease prevention (2), metabolic disorders (1), hyperlipidemia (1), pollution (1), and food safety (1)	Diabetes, heart failure, COPD, and cancer
5.3. What medical problems influence people’s working performance the most?	Joint disease (2), hypertension (2), chronic disease (2), diabetes (2), cardiac disease (1), bone disease (1), stroke (1), cancer (1), and rheumatoid arthritis (1)	COPD (3), hypertension (3), stroke (2), asthma (2), diabetes (2), arthritis (2), joint degeneration (1), infections (1), bronchitis (1), rash (1), gastric intestinal disorders (1), TB (1), mental disease (1), renal failure (1), psychological problems (1), and epilepsy (1)	Heart failure, COPD, arthritis, and severe back pain
5.5. What are the most commons patient diagnoses that you cannot treat at your own hospital and therefore, must refer to another hospital?	HIV (3), cardiac conditions (3), TB (2), hypertension (2), acute trauma (2), acute surgery (1), stroke (1), COPD (1), kidney stones (1), cancer (1), sepsis (1), children seizures (1), dengue fever (1), diabetic complications (1), malaria (1), and cataracts (1)	AMI (5), sepsis (2), stroke (2), head trauma (2), trauma (general) (1), cardiovascular diseases (1), dengue fever (1), endocrinology diseases (1), femur fracture (1), metabolic disease (1), severe asthma (1), severe heart failure (1), acute renal failure (1), hemorrhage (1), acute psychotic episodes (1), eye disorders (1), pregnancy complication (1), spinal trauma (1), cancer (1), epilepsy (1), GI issues (1), mental disorders (1), and drug-resistant asthma (1)	HIV, TB, psychiatric disorders, head trauma, and severe AMI

Abbreviations: AMI, acute myocardial infarction; COPD, chronic obstructive pulmonary disease; GI, gastrointestinal; HIV, human immunodeficiency virus; TB, tuberculosis. Footnotes: ^a^ Director is not aware of any neglected medical problems in the community.

**Table 4:** Acute medical response in the case of an emergency or natural disaster.

Survey Questions	Level 4 Community Health Centers N = 7 (Response Frequency by Sites Surveyed)	Level 3 District Hospitals N = 7 (Response Frequency by Sites Surveyed)	Level 2 Provincial Hospital N = 1
3.5. Does your hospital provide local patients with a pick-up using an ambulance in case of emergencies or natural disasters (e.g., floods, earthquakes, storms)?	No ambulances available (7)	Yes (7)	Yes, but only for natural disasters, not for patient emergencies
3.32 If your hospital lacks important diagnostic tests during emergencies or natural disasters where do you normally seek them?	District hospital (4), Hue Central Hospital (4), and Hue University Hospital (2)	Hue Central Hospital (4), Hue University Hospital (2), MEDIC Medical Center (1), and in case of emergencies/natural disasters people go to the closest hospital near their homes (1)	Hue Central Hospital, Ho Chi Minh City, Hanoi
5.4 In case of emergencies, where do the people turn to receive healthcare delivery?	District hospital (4), Hue University Hospital (4), and Hue Central Hospital (4)	DH (3), Hue University Hospital (2), Hue Central Hospital (2), and closest/nearest to the patient (1)	Trauma and injury severity dictate where patient goes; in cases of acute myocardial infarction or stroke, patients are allowed to decide where to seek medical attention

**Table 5:** Therapeutic turnaround time for tests sent to referral laboratories.

Survey Questions	Level 2 Provincial Hospital (N = 1)	Level 3 District Hospitals (N = 7) (Response Frequency by Sites Surveyed)	Level 4 Community Health Centers (N = 7) (Response Frequency by Sites Surveyed)
Tests sent to an outside referral laboratory ^a^	L-lactate, anti-CCP, NSE, and SPEP	HIV (4); dengue fever (4); malaria (3); TB (2); chlamydia (1); organisms that cannot be cultured (1 ^e^); and none, do not transfer patients for tests (1)	No tests available to perform, patient is transferred (6); HIV (1); and pap smears (1)
Name of referral laboratory ^b^	Hue Central Hospital ^d^	Center for Disease Control of Thua Thien Hue Province (4); Thua Thien Hue Center of HIV/AIDS Control (3); Hue University Hospital (1); Hue Central Hospital (1) and Third party private laboratory (1); TB and Pulmonary Disease Hospital (1); and none, do not transfer patients’ tests (1)	No tests available to perform, patient is transferred to the nearest district hospital (6); and nearest district hospital (1)
Time taken to receive results from referral laboratory ^c^	If delivered same morning, results are received in the afternoon; if delivered in the afternoon, the results are received the following day (7–24 h)	7–10 days (4); 1–3 days (2); 30 days (2); 7 h (1); and none, do not transfer patient tests (1)	No tests available to perform, patient is transferred (6); and 3–10 days (1)

Abbreviations: anti-CCP, anti-cyclic citrullinated peptide antibody test; HIV, human immunodeficiency virus; NSE, neuron specific enolase; SPEP, serum protein electrophoresis; TB, tuberculosis. Footnotes: ^a^ Survey question 4.21. ^b^ Survey question 4.22. ^c^ Survey question 4.23. ^d^ Biochemistry laboratory in Hue Central Hospital. ^e^ Typically, if a test cannot be performed, the patient is transferred downstream to a Level 1 or Level 2 hospital.

**Table 6:** Site-specific placement of POC resources in primary care centers, patient homes, and emergency medical services.

Hospital Responses ^a^	Primary Care Centers	Patient Home	Emergency Medical Services ^b,c^
Level 2 Provincial Hospital (N = 1)	ABO blood typing, blood gas, cardiac biomarkers, CBC, ECG, electrolytes, glucose meter, hyperlipidemia, ultrasound, and SpO_2_	Glucose meter and SpO_2_	Blood gas, CBC, glucose meter, electrolytes, and SPO_2_
Level 3 District Hospitals (N = 7) (Response frequency by sites surveyed)	Glucose meter (6), CBC (4), ECG (4), urinalysis (4), cardiac biomarkers (2), hyperlipidemia (2), malaria Ag (2), HBsAg (2), ultrasound (2), AST (2), ALT (2), bhCG (1), urea (1), creatinine (1), SpO_2_ (1), HPV (1), *H. pylori* (1), HIV (1), coagulation tests (1), prothrombin time (1), activated partial thromboplastin time (1), albumin (1), and bilirubin (1)	Glucose meter (7), BP (6), CBC (1), and electrolytes (1)	Glucose meter (4), ECG (2), cardiac biomarkers (2), BP (1), coagulation tests (1), ultrasound (1), and blood gas (1)
Level 4 Community Health Centers (N = 7) (Response frequency by sites surveyed)	Glucose meter (6), CBC (5), urinalysis (5), ECG (4), ultrasound (4), blood gas (3), HIV (2), coagulation tests (2), bhCG (2), ABO blood typing (1), cardiac biomarkers (1), BP (1), SpO_2_ (1), prothrombin time (1), AST (1), ALT (1), activated partial thromboplastin time (1), albumin (1), bilirubin (1), urea (1), creatinine (1), and spirometer (1)	Glucose meter (7) and BP (5)	Blood gas (2), ECG (2), cardiac biomarkers (2), malaria Ag (1), BP (1), and CBC (1)

Abbreviations: ALT, alanine aminotransferase; AST, aspartate aminotransferase; bhCG, beta-human chorionic gonadotropin; BP, blood pressure; CBC, complete blood cell count; ECG, electrocardiograph; HBsAg, hepatitis B surface antigen test; HIV, human immunodeficiency virus; HPV, human papillomavirus; *H. pylori*, helicobacter pylori; malaria Ag, malaria surface antigen test; SpO_2_, peripheral capillary oxygen saturation. Footnotes: ^a^ Survey questions 2.16, 2.17, 2.18, 2.24, and 4.25. ^b^ Five community health centers’ responses were provided. ^c^ Four district hospitals’ responses were recorded, and the remaining three district hospitals did not request for POC tests in the hands of EMS staff due to the current stepwise patient triage.

**Table 7:** Hospital preparedness for patients with highly infectious diseases.

Survey Questions	Level 2 Provincial Hospital (N = 1)	Level 3 District Hospital (N = 1)	Level 4 Community Health Center (N = 1)
6.1. Does your hospital have preparations in place for highly infectious disease outbreaks/epidemics?	No	No	No
6.5 Are staff trained to use PPE? If yes, which staff members?	Yes (Doctors, nurses, and lab staff)	Yes (Doctors, nurses, and lab staff)	Yes (Doctors and nurses)
6.6. Is there an Isolation unit or facility near/outside the hospital for patients suspected having a highly infectious disease?	No	No	No
6.7. Is there an isolation laboratory available for patients suspected of having a highly infectious agent?	Yes	No	No
6.12. Are there molecular diagnostics available to detect any highly infectious disease?	Yes (PCR to detect TB)	No	No
6.13. Is there proper isolation/quarantine equipment and facility to successfully contain a highly infectious disease?	No	No	No

Abbreviations: EVD, Ebola virus disease; PCR, polymerase chain reaction; PPE, personal protective equipment; TB, tuberculosis.

**Table 8:** Hospital preparedness for patients with Ebola virus disease.

Survey Questions	Level 2 Provincial Hospital (N = 1)	Level 3 District Hospital (N = 1)	Level 4 Community Health Center (N = 1)
Is your hospital prepared for a patient suspected of having EVD?	No	No	No
Does your hospital have diagnostic tests to detect EVD?	No	No	No
Does your hospital have PPE for staff when dealing with EVD?	Yes	Yes	Yes
What is the first step taken to isolate or quarantine a patient with EVD?	None, inform Hue Central Hospital (Level 1 ^a^)	None, inform the referral hospital	None, inform the referral hospital
Is the EVD patient transferred to a referral hospital?	Yes	Yes	Yes
What method of transportation is used to transfer the patient infected with EVD?	Ambulance	Ambulance	Ambulance ^b^
What is the first step taken to detect and diagnose a patient suspected of having EVD?	None	None	None

Abbreviations: EVD, Ebola virus disease; PPE, personal protective equipment. Footnotes: ^a^ Infectious disease department located in Hue Central Hospital. ^b^ Community health centers do not have ambulances on-site. Instead, the CHCs call 115, and an emergency medical service ambulance will drive to the CHC site to pick up a patient and then transfer the patient to a referral hospital.

**Table 9:** Government directives, test clusters, and available testing locations for patients with highly infectious diseases.

Disease	Test Clusters	Available Test Locations	Government Directives
Novel coronavirus disease 2019 (COVID-19)	● RT-PCR	● Levels 1 and 2	Decision 250/QD-BYT Directive for the diagnosis and treatment of COVID-19, 28 January 2022
	● Rapid antigen	● Levels 1, 2, 3, and 4	
Zika	● IgM ^a^	● Not available	Decision no. 439/QD-BYT Directive for the diagnosis and treatment of Zika, 5 February 2016
	● RT-PCR ^b^	● Not available ^i^	
	● Fetal ultrasound ^c^	● Levels 1, 2, and 3	
Dengue fever	● Rapid test ^d^: NS1, IgM	● Levels 1, 2, and 3	Decision no. 458/QD-BYT Directive for the diagnosis and treatment of dengue fever, 16 February 2011
	● ELISA ^e^: IgM, IgG	● Levels 1 and 2	
	● PCR ^f^	● Levels 1 and 2	
	● Viral culture ^f^	● Not available	
Middle East respiratory syndrome coronavirus (MERS-CoV)	● RT-PCR	● Not available ^i^	Decision no. 3014/QD-BYT Directive for the diagnosis and treatment of MERS-CoV, 13 August 2014
Ebola virus disease (EVD)	● Antigen	● Not available	Decision no. 2968/QD-BYT Directive for the diagnosis and treatment of Ebola Virus Disease, 8 August 2014
	● Antibody	● Not available	
	● PCR	● Not available ^i^	
	● Viral culture	● Not available	
Malaria	● Giemsa blood smear test	● Levels 1, 2, 3, and 4	Decision no. 4845/QD-BYT Directive for the diagnosis and treatment of malaria, 8 September 2016
	● Rapid diagnostic tests	● Levels 1, 2, 3, and 4	
Syphilis	● Dark field microscopy	● Levels 1, 2, and 3	Decision no. 75 QD/BYT Directive for the diagnosis and treatment of dermatologic diseases, pg. 190–191, 13 January 2015
	● Nonspecific tests (RPR and VDRL)	● Levels 1 and 2	
	● Specific tests (TPI, FTA, FTA-ABS, and TPHA)	● Levels 1 and 2	
Cholera	● Fecal smear	● Levels 1, 2, and 3	Decision no. 4178/QD-BYT Directive for the diagnosis and treatment of cholera, 21 October 2017
	● Fecal culture	● Levels 1, 2, and 3	
	● PCR	● Not available ^j^	
Human immunodeficiency virus (HIV)	● HIV tests ^g^	● Levels 1, 2, and 3	Decision no. 3047 QD/BYT Directive for the management, treatment, and care of HIV/AIDS, pg. 89–91, 22 July 2015
	● PCR ^h^	● Not available ^j^	
Tuberculosis	● Chest X-ray	● Levels 1, 2, and 3	Decision no. 4263 QD/BYT Directive for the diagnosis, treatment, and prophylaxis of tuberculosis, pg. 1–2, 13 October 2015
	● AFB test	● Levels 1, 2, and 3	
	● GeneXpert MTB/RIF	● Not available ^k,l^	
	● Sputum culture	● Levels 1, 2, and 3	
Methicillin- resistant staphylococcus aureus (MRSA)	● Bacterial culture-sensitivity test	● Levels 1, 2, and 3	None available
Severe acute respiratory syndrome (SARS)	● No specific test	● Not applicable	Decision no. 1113/2003/QD/BYT Directive for the diagnosis, treatment, and prophylaxis of SARS, 4 April 2003

Abbreviations: AFB, acid-fast bacteria; CBC, complete blood cell count, ELISA, enzyme-linked immunosorbent assay; FTA/FTA-ABS, fluorescent treponemal antibody absorption; MTB/RIF, mycobacterium tuberculosis resistance to rifampicin; NS1, nonstructural protein-1; RPR, rapid plasma regain; RT-PCR, reverse transcription polymerase chain reaction; TB, tuberculosis; TPHA, treponema pallidum hemagglutination assay; TPI; treponema pallidum immunization; VDRL, venereal disease research laboratory. Footnotes: ^a^ Performed four days after the onset of symptoms. ^b^ Test chosen for Zika diagnosis with specimens of either serum, urine, CSF, or amniotic fluid. ^c^ Performed in infected pregnant women in order to detect microcephaly in the fetus. ^d^ Detect NS1 antigen within the first five days; detect IgM antibody after the fifth day. ^e^ Detect IgM: taken after the fifth day; detect IgG: taken 2 times, 1 week apart. If the second reading is 4 times the value of the first test, then patient is ruled as positive for having dengue fever. ^f^ Viral isolation from blood sample collected when patient is suffering from a fever. ^g^ For children older than 18 months old and adults. Adults are diagnosed on the basis of HIV antibody testing: having HIV seropositive for all three HIV antibody tests from different types of biological agents as described by the Ministry of Health. ^h^ For children <18 months old. ^i^ Suspected cases are sent and are confirmed in centers which are verified by WHO and are authorized by the Ministry of Health (National Hospital of Tropical Disease, Hospital of Tropical diseases in Ho Chi Minh City, National Institute of Hygiene and Epidemiology, and Pasteur Institute in Ho Chi Minh City). ^j^ No reagents are available to perform the test. ^k^ GeneXpert MTB/RIF is available at the TB and Respiratory Disease Center; however, it is unavailable at any hospital level. ^l^ Hue University Hospital has PCR instrument to detect TB.

**Table 10:** Emerging pandemic and epidemic infectious disease threats and diagnostic tests available in Central Vietnam hospitals.

Disease ^a^	Level 4 Community Health Centers N = 7 (Availability %, Method)	Level 3 District Hospitals N = 7 (Availability %, Method)	Level 2 Hospital N = 1 (Availability, Method)
COVID-19	100, RAgTs	100, RAgTs; 14, PCR	Yes, RT-PCR and RAgT
Zika	0	0	No
Ebola	0	0	No
MERS-CoV	0	0	No
SARS	0	0	No
Influenza A	0	0	Yes, PCR
Influenza B	0	0	Yes, PCR
H1N1	0	0	Yes, PCR
H7N9	0	0	Yes, PCR
**Bloodborne Pathogens and Direct and Indirect Contact Transmission**			
HIV	0	100, rapid antibody test	Yes, rapid antibody test
Syphilis	0	14, dark field microscopy	Yes, rapid antibody test, RPR, and TPHA
Gonorrhea	0	14, gram-stained smear	Yes, real-time PCR, rapid antibody test, gram stain, and culture
Chlamydia	0	0	Yes, PCR and rapid antibody test
HBV	0	100, antigen and antibody	Yes, real-time PCR and antigen/antibody
HCV	0	43, antibody	Yes, antibody
HPV	0	14, pap smear	Yes, PCR and pap smear
CMV	0	0	Yes, PCR, antibody
HSV	0	0	Yes, PCR and Tzanck smear
**Digestive and Foodborne Illnesses**			
Dysentery	0	14, culture	Yes, culture
HAV	0	29, antibody	Yes, antibody
*H. pylori*	0	29, antibody, breath test, and culture	Yes, antibody, breath test, and culture
*E. coli*	0	14, culture	Yes, culture
Salmonella	0	14, culture	Yes, culture
Toxoplasma	0	0	Yes, antibody
**Vector-Borne Infectious Diseases**			
Dengue fever	0	29, NS1 and antibody	Yes, NS1 and antibody
Malaria	29, blood culture and rapid antibody test	43, blood culture and rapid antibody test	Yes, blood culture and rapid antibody test
**Re-Emerging Airborne Infectious Diseases**			
TB	0	14, AFB	Yes, PCR, AFB, and culture
Rubella	0	14, rapid antibody test	Yes, rapid antibody test

Abbreviations: AFB, acid-fast bacillus test; AIDS, acquired immunodeficiency syndrome; CMV, cytomegalovirus; Ebola, Ebola virus disease; *E. coli*, *Escherichia coli*; HAV, hepatitis A virus; HBV, hepatitis B virus; HCV, hepatitis C virus; HIV, human immunodeficiency virus; HPV, human papillomavirus; HSV, herpes simplex virus; *H. pylori*, helicobacter pylori; H1N1, swine flu; H7N9, avian influenza A; MERS-CoV, Middle East respiratory syndrome coronavirus; NS1, nonstructural protein 1 antigen test; PCR, polymerase chain reaction; RAgT, rapid antigen test; RPR, rapid plasma reagin; SARS, severe acute respiratory syndrome; TB, tuberculosis; TPHA, treponema pallidum hemagglutination assay; Zika, Zika virus disease. Footnotes: ^a^ Survey questions 4.14, 4.15, and 6.12 (see infectious disease survey questionnaire).

## Supplementary Materials

The following supporting information can be downloaded at: https://www.mdpi.com/article/10.3390/diagnostics12092047/s1. File S1: Short version on-site investigator survey questionnaire, Central Vietnam. Point-of- care testing, rapid response, and Spatial Care Paths^TM^ for acute myocardial infarction and other medical challenges (In English). File S2: Long version on-site investigator survey questionnaire, Central Vietnam. Point-of- care testing, rapid response, and Spatial Care Paths^TM^ for acute myocardial infarction and other medical challenges (In English). File S3: Long version on-site investigator survey questionnaire, Central Vietnam. Point-of- care testing, rapid response, and Spatial Care Paths^TM^ for acute myocardial infarction and other medical challenges (In Vietnamese). File S4: Infectious disease survey questionnaire (in English). File S5: Infectious disease survey questionnaire (in Vietnamese).
