# Supplementary material for: Optimizing Public Health Preparedness for Highly Infectious Diseases in Central Vietnam
Source: Diagnostics (Basel). 2022 Aug 24;12(9):2047. doi: 10.3390/diagnostics12092047 (PMC9497681; doi:10.3390/diagnostics12092047)
Supplement: Supplementary file 1 [file diagnostics-12-02047-s001.zip › S2-Survey_Long Questionnaireí¬Central Vietnam FINAL VERSION.pdf]

ON-SITE INVESTIGATOR SURVEY QUESTIONNAIRE, CENTRAL VIETNAM

"POINT-OF-CARE TESTING, RAPID RESPONSE, AND SPATIAL CARE PATHS™ FOR ACUTE MYOCARDIAL INFARCTION AND OTHER MEDICAL CHALLENGES

In collaboration with University of Hue and Hue University of Medicine and Pharmacy

Research Team: Point-of-Care Testing Center for Teaching and Research (POCT•CTR)

School of Medicine, University of California, Davis, USA 95616

Gerald J. Kost, MD, PhD, MS, FACB; Director and Principal Investigator

July 2017 (20 pages)

Goal: To design point-of-care testing (e.g., on-site cardiac biomarker testing for cTnI, cTnT, & BNP) to improve patient outcomes in the region surrounding Hue University of Medicine and Pharmacy.

TABLE OF CONTENTS **PAGE**

Topic I. Demography……………………………………………………….………2

Topic II. Point-of-Care Testing (POCT) …………………………………………3

Topic III. Critical Care and Patient Access………………………………………7

Topic IV. Clinical Laboratory……………………………………………..………14

Topic V. Medical Problem Solving in the Community…………..……………19

Accession # ....(< Please fill in the serial number or code for the site visited.)

Surveyor(s) ………………………….…(< Team members contributing to these data.)

TOPIC I. DEMOGRAPHY

1.1 Type of hospital ( ) Community hospital ( ) Regional hospital ( ) University hospital
( ) Private hospital ( ) Military hospital
( ) Other..

1.2 Size of hospital ( ) Small ( ) Medium ( ) Large

1.3 Number of beds......................

1.4 Number of physicians...........................

1.5 Number of pharmacists........................

1.6 Number of nurses.............................

1.7 Number of laboratory personnel: Medical technologist.......; Medical science staff ....

1.8 Number of ambulances....................

1.9 Is there an emergency room (ER)?

( ) Yes ( ) No ( ) Don't know

1.10 Are there operating rooms (ORs)?

( ) Yes, identify number of ORs .... ( ) No ( ) Don't know

1.11 Are there intensive care units (ICUs)?

( ) Yes ( ) No ( ) Don't know

- If so, identify number of ICUs...............rooms

1.12 Are there neonatal intensive care units (NICUs)?

( ) Yes ( ) No

- If so, identify number of NICUs.................rooms

1.13 Are there cardiac care units (CCUs)?

( ) Yes ( ) No

- If so, identify number of CCU beds..........................

1.14 Is there a labor room (LR)?

- ( ) Yes ( ) No

1.15 Has the hospital been certified by ISO, The Joint Commission (TJC), Government, or other accreditation agency(ies), such as the College of American Pathologists (CAP)?

( ) ISO ( ) TJC ( ) CAP ( ) Government, specify ________________

( ) Other , specify..

TOPIC II. POINT-OF-CARE TESTING (POCT)

DEFINITION: Point-of-care (POC) testing is testing *at or near the site of patient care*.

POC testing includes near-patient testing, bedside testing, and satellite laboratories

Questions regarding the uses and objectives of POC tests and devices follow.

2.1 Is there a point-of-care (POC) testing or bedside testing program?

( ) Yes ( ) No

2.2 Is there a POC testing director?

( ) Yes ( ) No

2.3 Is there a POC Coordinator, that is, personnel who manage and oversee the POCT program?

( ) Yes ( ) No

2.4 List the tests performed at the patient bedside or near the patient in the following departments:

Ward (a large room in a hospital where a number of patients often needing similar treatment are cared for)...

OR..

ER..

LR..

ICU..

NICU...

CCU...

Other..

2.5 Specify the POC tests or devices that your hospital does NOT, but would like to perform in the following departments:

Ward..

OR..

ER..

LR..

ICU..

NICU..

CCU..

Other..

2.6. Please rank the POC tests that you need the most and where the diagnostic tests should be located (e.g., patient homes, primary care, clinics, health promoting hospital, emergency room, labor and delivery, intensive care unit, ward, or ambulance).

**Diagnostic tests**	**Rank**	**Where to place**
Blood typing (A, B, O, Rh)	_____	_____________
Rapid microbiology tests	_____	_____________
Coagulation (PT/ INR)	_____	_____________
Blood gases (pH, pO_2, pCO_2)	_____	_____________
Chemistry/ electrolytes/ ionized calcium	_____	_____________
Hematology (CBC, differential, platelet)	_____	_____________
Cardiac biomarkers (cTnT, cTnI, others)	_____	_____________
Pulse oximeter (O_2 saturation)	_____	_____________

2.7. For POC testing, rank the **top five diagnostic tests** from most useful (1) to least useful (5):

1. (most useful) _____________ 2. _____________ 3. _____________
4. _____________ 5. _____________

2.8. Specify and rank POC tests/ devices used most frequently in your **ER.**

1. _____________ 6. _____________
2. _____________ 7. _____________
3. _____________ 8. _____________
4. _____________ 9. _____________
5. _____________ 10. _____________

2.9 Specify and rank POC tests/ devices used most frequently in your **ICU.**

1. _____________ 6. _____________
2. _____________ 7. _____________
3. _____________ 8. _____________
4. _____________ 9. _____________
5. _____________ 10. _____________

2.10 Specify and rank POC tests/ devices used most frequently in your **OR.**

1. _____________ 6. _____________

2. ______________ 7. ______________
3. ______________ 8. ______________
4. ______________ 9. ______________
5. ______________ 10. ______________

2.11 Specify and rank POC tests/ devices used most frequently in your **NICU.**
1. ______________ 6. ______________
2. ______________ 7. ______________
3. ______________ 8. ______________
4. ______________ 9. ______________
5. ______________ 10. ______________

2.12 Specify and rank POC tests/ devices used most frequently in your **CCU.**
1. ______________ 6. ______________
2. ______________ 7. ______________
3. ______________ 8. ______________
4. ______________ 9. ______________
5. ______________ 10. ______________

2.13 Specify and rank POC tests/ devices used most frequently in your **LR.**
1. ______________ 6. ______________
2. ______________ 7. ______________
3. ______________ 8. ______________
4. ______________ 9. ______________
5. ______________ 10. ______________

2.14 Specify and rank POC tests/ devices used most frequently in your **ward.**
1. ______________ 6. ______________
2. ______________ 7. ______________
3. ______________ 8. ______________
4. ______________ 9. ______________
5. ______________ 10. ______________

2.15 Specify and rank POC tests that are frequently used in your **ambulances.**
1. ______________ 6. ______________
2. ______________ 7. ______________
3. ______________ 8. ______________
4. ______________ 9. ______________
5. ______________ 10. ______________

2.16 Which POC tests would you like to place in local **primary care centers, clinics, or health promoting hospitals** to improve patient access to diagnosis? List and rank:
1. ______________ 6. ______________
2. ______________ 7. ______________

3. ______________ 8. ______________
4. ______________ 9. ______________
5. ______________ 10. _____________

2.17 Which POC tests would you place in **patient homes**, so they can do their own monitoring and call in, email, or directly transmit via Internet results to you or a physician? List and rank:

1. ______________ 6. ______________
2. ______________ 7. ______________
3. ______________ 8. ______________
4. ______________ 9. ______________
5. ______________ 10. _____________

2.18 Suppose a patient is suddenly critically ill at home. Which POC tests should **Emergency Medical Staff** perform while transporting the patient to speed up the treatment. List and rank:

1. ______________ 6. ______________
2. ______________ 7. ______________
3. ______________ 8. ______________
4. ______________ 9. ______________
5. ______________ 10. _____________

2.19 Who mainly performs the POC testing?

( ) Physician ( ) Nurse ( ) Lab staff ( ) Other........................

2.20 Do patients themselves perform testing or monitoring at their sites of patient care?

( ) Yes ( ) No

If so, which ones (list)……………………………………….

2.21 After they leave the hospital, do patients perform testing at their homes?

( ) Yes ( ) No

- If so, what is that test? Please specify…………………

2.22 Do patients consult physicians about the test results produced by themselves?

( ) Yes ( ) No

2.23 Do patients or their families receive training on how to perform testing themselves?

( ) Yes ( ) No

2.24 What types of additional POC tests should be provided to your hospital?

Please specify……………………………………………………………………………

2.25 What types of additional tests should be provided for patients to perform themselves?

Please specify……………………………………………………………………………

2.26 To facilitate patient access to healthcare delivery, where should POC resources be placed in your health network?

( ) At patients' home, allowing them to perform self-testing
( ) At a primary care site or in a health promoting hospital close to patient homes and work
( ) At a hospital close to where patients live, and if so, how close _______ (km)
( ) At a regional hospital, and if so, at what maximum distance from homes ____ (km)
( ) Other, specify…………………………………………..

TOPIC III. CRITICAL CARE AND PATIENT

- **Ambulance Transport**

3.1 Do you have helicopter transport?
( ) Yes, specify the frequency per month: ………. ( ) No

3.2 How far can your ambulance travel to pick up a patient?
Please specify distance…………………(km) and location(s)…………………………

3.3 If patients were 5km, 10 kms, 25 kms, and 50 km away approximately how long would it take to reach them and bring them back?

Distance	Amount of time (min)	Actual pick-up site
5 kms		
10 kms		
25 kms		
50 kms		

3.4 How often does you EMS team transport patients using an ambulance?

………………a day ………………a month ………………a year

3.5 Does your hospital provide local patients with a pick-up using an ambulance in case of emergencies or natural disasters (e.g., floods, earthquakes, storms)?

( ) Yes ( ) No

3.6 Do you need to transport **critically ill patients** to other hospitals?

( ) Yes, specify transport distance ____ (km) and referral site _______ ( ) No

If yes, why…………………………………………………………………………….

If no, why not………………………………………………………………………….

3.7 If it is necessary to transfer patients to other hospitals, where do you transfer them and how long does it take?

Name of referral hospital	Amount of time (min)

3.8 Identify the community hospital closest to your hospital...

- Specify the distance…………………km.
- Specify the travel time……………….min.

3.9 Identify the referral (or regional) hospital closest to your hospital................................

- Specify the distance…………………km.
- Specify the travel time……………….min.

3.10 Please specify the number of primary care sites, primary care clinics, health promoting hospitals (HPH), or primary care units (PCUs) that are your hospital's responsibility:

**Name**	**Distance (km)**	**Travel time (min)**	**Personnel**			
			**Physicians**	**Working hours**	**Nurses**	**Working hours**
1.						
2.						
3.						
4.						
5.						
6.						
7.						
8.						

9.							
10.							

3.11 Now describe ambulance sites covered from your hospital. State distances, transport times, and destinations.

Site (pick up) **Distance (km)** **Transport time (min)** **Destination**

1. ____________ → ____________ → ____________ → __________
2. ____________ → ____________ → ____________ → __________
3. ____________ → ____________ → ____________ → __________
4. ____________ → ____________ → ____________ → __________
5. ____________ → ____________ → ____________ → __________
6. ____________ → ____________ → ____________ → __________
7. ____________ → ____________ → ____________ → __________
8. ____________ → ____________ → ____________ → __________
9. ____________ → ____________ → ____________ → __________
10. ____________ → ____________ → ____________ → __________

3.12 How many patients visit your hospital each day? ………………………………

What percent have acute illness? ………………….%

What percent have chronic illness? ………………%

- **Cardiac Event (e.g., acute myocardial infarction)**

3.13 Are there health facilities with which you interact for cardiac care? Please fill out the details below. [CH, community hospital; RH, regional hospital]

Name	Type (CH, RH, other)	Distance (km)	Time (min)

3.14 What are the top five facilities and/or technologies you would like to have for cardiac diagnostic support?

No	Describe
1	
2	
3	
4	
5	

3.15 Do you have cardiac care specialists, such as interventional cardiologists?

If so, please describe:……………………………………………..

3.16 What diagnostic technology do you use to support the diagnosis of acute myocardial infarction (AMI)? ( ) cTnI ( ) cTnT ( ) Other biomarkers:…………………………

3.17 If you obtain a positive diagnosis of AMI, can you rapid provide interventional care?
( ) Yes, response time:……………… ( ) No

- If not, where would they travel for care?
- What is the travel distance (km)?_____
- What is the travel time (min)?_____

3.18 What are the next steps if you have a positive diagnosis for the following diseases/ conditions?

Diseases/ conditions	Next steps
Acute Myocardial Infarction	
Acute trauma	
Acute surgeries	
Diabetes Mellitus (Diabetes Ketoacidosis)	
Hypertension	
Stroke	
Asthma	
Sepsis	
Respiratory symptoms	
Digestive symptoms	
Disorders of urethra & urinary tract	
Infectious diseases	
HIV/ sexual transmitted diseases	
Other(s)………………………………………………	

3.19 If the next steps are to transfer to another hospital for referral care, to which hospital(s) do you transfer, how long does it take, and how far away is it?

Diseases/ conditions	Referral hospital	Amount of time (min)	Distance (km)
Acute Myocardial Infarction			
Acute trauma			
Acute surgeries			
Diabetes Mellitus (Diabetes Ketoacidosis)			
Hypertension			
Stroke			
Asthma			
Sepsis			
Respiratory symptoms			

Digestive symptoms			
Disorders of urethra & urinary tract			
Infectious diseases			
HIV/ sexual transmitted diseases			
Other(s)…………………………………………………			

- **Diabetes Mellitus**

3.20 Where is glucose screening test performed?

( ) Home ( ) Local community ( ) Closest community hospital

( ) Health promoting hospital or primary care unit ( ) Other......................

3.21 Who does glucose screening test?

( ) Physician ( ) Nurse ( ) Public health staff ( ) Patient

( ) Other, specify................................

3.22 What type of specimen is used for glucose testing?

( ) Capillary Blood ( ) Plasma ( ) Serum ( ) Other................................

3.23 Who interprets the result of glucose testing?

( ) Physician ( ) Nurse ( ) Public health staff ( ) Patient

( ) Other....................................

3.24 What cut-offs in mg/dL do you use for **prediabetes**?

From……… mmol/L to………… mmol/L

3.25 What cut-offs in mg/dL do you use for **diabetes**?

From……… mmol/L to………… mmol/L

3.26 Is there a diabetic screening program in a community?

( ) Yes ( ) No

- If so, where is the screening test done?

( ) Local community ( ) Closest community hospital

( ) Health promoting hospital ( ) Other......................

3.27 Do patients use glucose meters to perform self-monitoring of blood glucose level?

( ) Yes ( ) No

If so, who pays for patients' glucose meters? Please specify……………………………

3.28 If the patient has complications (e.g., kidney failure, retinopathy), where is the patient treated? Please specify……………………………

- **Support of Diagnostic Tests**

3.29 For each of these conditions what diagnostic tests do you perform? Please fill in the tests for the following diseases/ conditions

Diseases/ conditions	Diagnostic test
Acute Myocardial Infarction	
Acute trauma	
Acute surgeries	
Diabetes Mellitus (Diabetes Ketoacidosis)	
Hypertension	
Stroke	
Asthma	
Sepsis	
Respiratory symptoms	
Digestive symptoms	
Disorders of urethra & urinary tract	
Infectious diseases	
HIV/ sexual transmitted diseases	
Other(s)………………………………………………	

3.30 What hospital would you find this type of support?

Diseases/ conditions	Name of hospital
Acute Myocardial Infarction	
Acute trauma	
Acute surgeries	
Diabetes Mellitus (Diabetes Ketoacidosis)	
Hypertension	
Stroke	
Asthma	
Sepsis	
Respiratory symptoms	
Digestive symptoms	
Disorders of urethra & urinary tract	
Infectious diseases	

HIV/ sexual transmitted diseases	
Other(s)………………………………………………	

3.31 What type of POC devices or test kits do you currently have for the following?

**Diseases/ conditions**	**POC device/ test kit**
Acute Myocardial Infarction	
Acute trauma	
Acute surgeries	
Diabetes Mellitus (Diabetes Ketoacidosis)	
Hypertension	
Stroke	
Asthma	
Sepsis	
Respiratory symptoms	
Digestive symptoms	
Disorders of urethra & urinary tract	
Infectious diseases	
HIV/ sexual transmitted diseases	
Other(s)………………………………………………	

3.32 In your health network, if your hospital lacks important diagnostic tests during emergencies or natural disasters where do you normally seek for them?

Please specify…………………………………………………………………….

- **Specialized Care**

3.33 For which health facilities does your hospital support if serving as a referral hospital?

Please specify…………………………………………………………………………..
……………………………………………………………………………………….

……………………………………………………………………………………….

3.34 From which health facilities does your hospital need to seek specialized healthcare?

Specialized healthcare	Health facility

3.35 In your health network, how would you improve access to care for cardiac arrest patients?

Please explain………………………………………………………………………………

………………………………………………………………………………………………

TOPIC IV. CLINICAL LABORATORY

- **Service Hours**

4.1 Working days Time:………………To………………..

Weekends/holidays Time:………………To………………..

On call service ( ) Yes ( ) No

4.2 Please list and rank twenty **diagnostic tests** that you need the most to care for your patients (1, most important; 20, least important):

Most Important

1. __________ 2. __________ 3. __________ 4. __________ 5. __________
6. __________ 7. __________ 8. __________ 9. __________ 10. _________
11. _________ 12. _________ 13. _________ 14. __________ 15. _________
16. _________ 17. _________ 18. _________ 19. __________ 20. _________ *Least important*

4.3 Please rank the following **laboratory sections** in order of importance (1, highest):

_____ Chemistry _____Molecular diagnostics
_____ Hematology _____ Cytology
_____ Microbiology _____ Noninvasive monitoring (pulse oximeter, oxygen saturation monitoring)
_____ Immunology _____ Other (write in the section name): ____________
_____ Microscopy _____ Other (write in): __________________________
_____ Blood Bank _____ Other (write in): __________________________

- **Number of Patients, Locations, and Healthcare Access**

4.4 How many patients do you see a day/ month/ year?

………………a day ………………a month ………………a year

4.5 Where are most of them located? Please specify the location……………………………

4.6 How do most of patients get to your facility?

Please specify the following:

………. % Walking

………. % Motorcycles

………. % Automobiles

………. % Public transportation

………. % Ambulances

………. % Helicopter

4.7 Please provide the estimated frequencies of visits for the following diseases/ conditions

Diseases/ conditions	Estimated frequency of visit
Acute Myocardial Infarction	
Acute trauma	
Acute surgeries	
Diabetes Mellitus (Diabetes Ketoacidosis)	
Hypertension	
Stroke	
Asthma	
Sepsis	
Respiratory symptoms	
Digestive symptoms	
Disorders of urethra & urinary tract	
Infectious diseases	
HIV/ sexual transmitted diseases	
Other(s)………………………………………………	

- **Oxygen Saturation Monitoring (Pulse Oximetry)**

4.8 Are there any oxygen saturation monitors (pulse oximeters)?

 ( ) Yes ( ) No

If **yes**, identify the number of oxygen saturation monitors ……………………..

4.9 Identify the number of oxygen saturation monitors available in each department:

ER ....

OR...............................

ICU............................. .

NICU...........................

CCU............................ .

LR............................... .

Ward........................... .

Ambulance...................

Other............................

- **Critical Care**

4.10 Is blood gas testing performed in the laboratory?

 ( ) Yes ( ) No

If so, which of the following measurements are performed in the laboratory?

pO_2 ( ) Yes ( ) No

pCO_2 ( ) Yes ( ) No

pH ( ) Yes ( ) No

HCO_3 ( ) Yes ( ) No

4.11 Are electrolytes determined in the laboratory?

 ( ) Yes ( ) No

- If so, please specify those electrolytes………………………………………………………

4.12 Who performs the following tests?

- Blood gases ( ) MD ( ) Nurse ( ) Lab staff ( ) Other…………………
- Electrolytes ( ) MD ( ) Nurse ( ) Lab staff ( ) Other…………………
- Ionized calcium ( ) MD ( ) Nurse ( ) Lab staff ( ) Other…………………

- O_2 □( ) MD □ ( ) Nurse □ ( ) Lab staff □ ( ) Other…………………
- pCO_2 □( ) MD □ ( ) Nurse □ ( ) Lab staff □ ( ) Other…………………
- pH □( ) MD □ ( ) Nurse □ ( ) Lab staff □ ( ) Other…………………
- HCO_3 ( ) MD ( ) Nurse ( ) Lab staff ( ) Other…………………

4.13 Does the laboratory perform bloodstream pathogen cultures? □

( ) Yes ( ) No

4.14 Which and where of the following tests are performed?

- Hepatitis A □ ( ) Yes ( ) No Where…………………
- Hepatitis B (HBsAg) ( ) Yes ( ) No Where…………………
- Hepatitis B (HB Ab) □ ( ) Yes ( ) No Where…………………
- Influenza A ( ) Yes ( ) No Where…………………
- Influenza B ( ) Yes ( ) No Where…………………
- H1N1 ( ) Yes ( ) No Where…………………
- H7N9 ( ) Yes ( ) No Where…………………
- Mers-CoV ( ) Yes ( ) No Where…………………
- HIV (AIDS) □ ( ) Yes ( ) No Where…………………
- Strep throat ( ) Yes ( ) No Where…………………
- Blood culture □ ( ) Yes ( ) No Where…………………
- Growing on culture plate ( ) Yes ( ) No Where…………………

4.15 Except for the tests in **4.14**, are there any other tests for infectious diseases?

……

……

- **Cardiovascular disease**

4.16 Which of the following tests are performed?

- CTn I (cardiac troponin I) ( ) Yes □ ( ) No
  If so, type: □ ( ) qualitative □ ( ) quantitative
- CTn T (cardiac troponin T) ( ) Yes □ ( ) No
  If so, type: □ ( ) qualitative □ ( ) quantitative

- CK ( ) Yes ( ) No
- CK-MB ( ) Yes ( ) No
- LD (LDH) ( ) Yes ( ) No
- AST ( ) Yes ( ) No
- Myoglobin ( ) Yes ( ) No
- BNP or NT-pro BNP ( ) Yes ( ) No
- Other...

- **Hematology**

4.17 Is there a complete blood count testing?

( ) Yes ( ) No

4.18 Is there a coagulation testing?

( ) Yes ( ) No

- If so, what types of tests are performed in the laboratory?

( ) PT ( ) aPTT ( ) PT and aPTT ( ) Other……………………….

4.19 Do the nurses perform any laboratory diagnostic tests the same as laboratory staff do?

( ) Yes ( ) No

- If so, please specify the laboratory tests the nurses can perform

 ( ) Hematology, specify…………………………………………………

 ( ) Serology, specify……………………………………………………

 ( ) Microbiology, specify……………………………………………….

 ( ) Chemistry, specify…………………………………………………..

 ( ) Urinalysis, specify…………………………………………………..

( ) Other, specify……………………………………………………

4.20 What any new instruments or tests does your laboratory still need?

……………………………………………………………………………………………………

4.21 What test(s) does your laboratory need to send to outside laboratory or referral laboratory?

……………………………………………………………………………………………………

4.22 Please specify the name and location of outside laboratory or referral laboratory

……………………………………………………………………………………………………

4.23 how long does it take to receive the testing results from the outside lab or referral lab (Identify turnaround time)

……………………………………………………………………………………………………

4.24 What types of tests are performed at health promoting hospitals?

 ( ) Glucose Test ( ) Pregnancy Test ( ) Urine Test

 ( ) Protein Test ( ) Hematocrit ( ) Other..............................

4.25 What new instruments or tests would help improve patients' health outcomes at health promoting hospitals?

....

....

4.26 Who directs the diagnosis testing at health promoting hospitals?

( ) MD ( ) Nurse ( ) Lab staff ( ) Public health staff ( ) Other.......................

......

4.27 Who is responsible for doing quality control of the diagnostic testing at health promoting hospitals?

( ) MD ( ) Nurse ( ) Lab staff ( ) Public health staff ( ) Other......................

TOPIC V. MEDICAL PROBLEM SOLVING IN THE COMMUNITY

5.1 Please list and rank the most common medical problems in your community (1, top; 10, least important)

1……………………………………… 6. ………………………………………

2……………………………………… 7. ………………………………………

3……………………………………… 8. ………………………………………

4……………………………………… 9. ………………………………………

5……………………………………… 10. ………………………………………

5.2 Which medical problems are neglected?

Please specify…………………………………………………………………………

5.3 What medical problems influence peoples' working performance the most?

Please specify…………………………………………………………………………………

5.4 In case of emergencies, where do the people turn to receive healthcare delivery?

Please specify…………………………………………………………………………………

5.5 What are the most common patient diagnoses that you cannot treat at your own hospital and therefore, must refer to another hospital? List and rank: (1, top; 5, least important)

1……………………………………….(most common)

2……………………………………….

3……………………………………….

4……………………………………….(least common)

5……………………………………….

5.6 For the Surveyor to fill in: Identify the **most important and highest impact improvements** that POC testing, when implemented, will produce at this site, based on actual survey evidence.

1.………………………………………………………………………………………………

………………………………………………………………………………………………

……………………………………………………………………………………………......

2………………………………………………………………………………………………

………………………………………………………………………………………………

……………………………………………………………………………………………

3………………………………………………………………………………………………

………………………………………………………………………………………………

……………………………………………………………………………………………

Version 10.4

July 7, 2017
