# Supplementary material for: Optimizing Public Health Preparedness for Highly Infectious Diseases in Central Vietnam
Source: Diagnostics (Basel). 2022 Aug 24;12(9):2047. doi: 10.3390/diagnostics12092047 (PMC9497681; doi:10.3390/diagnostics12092047)
Supplement: Supplementary file 1 [file diagnostics-12-02047-s001.zip › S3-Vietnamese Survey ĐIỀU TRA KHẢO SÁT VIÊN TAI CHỖ - GÓP Ý (ÂN).pdf]

PHIẾU KHẢO SÁT TAI CHỖ, MIỀN TRUNG VIỆT NAM

"XÉT NGHIỆM NHANH TẠI CHỖ, XÉT NGHIỆM NHANH, VÀ CHĂM SÓC THEO TUYẾN CHO NHỒI MÁU CƠ TIM CẤP TÍNH VÀ NHỮNG THÁCH THỨC Y KHOA KHÁC

Phối hợp với Đại học Huế và trường Đại học Y Dược Huế

Nhóm nghiên cứu: Trung tâm Giảng dạy và Nghiên cứu Xét nghiệm nhanh tại chỗ (POCT • CTR)

Trường Y, Đại học California, Davis, Hoa Kỳ 95616

T.S. BS. Gerald J. Kost, MS, FACB; Giám đốc và Điều tra viên chính

Tháng 7 năm 2017 (20 trang)

Mục tiêu: Thiết kế các xét nghiệm nhanh tại chỗ (ví dụ, xét nghiệm chỉ điểm tim mạch nhanh tại chỗ cho cTnI, cTnT, & BNP) để cải thiện kết quả điều trị của bệnh nhân trong vùng xung quanh Đại học Y Dược Huế.

MỤC LỤC **TRANG**

Chủ đề I. Nhân lực…….. 2

Chủ đề II. Xét nghiệm nhanh tại chỗ (POCT) 3

Chủ đề III. Chăm sóc tích cực và Tiếp cận Bệnh nhân 7

Chủ đề IV. Phòng xét nghiệm lâm sàng .... ……….. 14

Chủ đề V. Giải quyết vấn đề y khoa trong cộng đồng 19

Nhập # .... (<Vui lòng điền số seri hoặc mã cho vùng bạn đến thăm.)

Người khảo sát .... (<thành viên trong nhóm đóng góp vào những dữ liệu này.)

CHỦ ĐỀ I. NHÂN LỰC

1.1 Loại bệnh viện ( ) BV cộng đồng ( ) BV khu vực ( ) BV Đại học
( ) BV tư nhân ( ) BV Quân đội
( ) Khác ....

1.2 Kích thước của bệnh viện ( ) Nhỏ ( ) Trung bình ( ) Lớn

1.3 Số giường ....

1.4 Số Bác sĩ ....

1.5 Số Dược sĩ ....

1.6 Số Điều dưỡng ....

1.7 Số Nhân viên phòng XN: Kỹ thuật viên..............; Cử nhân khoa học y khoa ....

1.8 Số xe cứu thương ....

1.9 Có phòng cấp cứu (ER) không?

( ) Có ( ) Không ( ) Không biết

1.10 Có phòng mổ (OR) không?

( ) Có, xác định số lượng phòng mổ.................. ( ) Không ( ) Không biết

1.11 Có các đơn vị chăm sóc tích cực (ICU) không?

( ) Có ( ) Không ( ) Không biết

- Nếu vậy, xác định số ICU ....phòng

1.12 Có các đơn vị chăm sóc tích cực sơ sinh (NICU) không?

( ) Có ( ) Không

- Nếu vậy, xác định số NICU .... phòng

1,13 Có đơn vị chăm sóc tim mạch (CCU)?

( ) Có ( ) Không

- Nếu vậy, xác định số lượng giường CCU..................

1,14 Có phòng sinh (LR)?

- ( ) Có ( ) Không

1.15 Bệnh viện có được chứng nhận bởi ISO, Hội đồng liên kết (The Joint Commission (TJC)), Chính phủ, hoặc các cơ quan công nhận khác, chẳng hạn như Hiệp hội Bệnh lý Hoa kỳ (College of American Pathologists (CAP))?

( ) ISO ( ) TJC ( ) CAP ( ) Chính phủ, xác định......................

( ) Khác, chỉ ra..

CHỦ ĐỀ II. XÉT NGHIỆM NHANH TẠI CHỖ (POCT)

ĐỊNH NGHĨA: Xét nghiệm nhanh tại chỗ (POC) là xét nghiệm *tại hoặc gần khu vực chăm sóc bệnh nhân.*

Xét nghiệm nhanh tại chỗ bao gồm xét nghiệm gần bệnh nhân, xét nghiệm tại giường bệnh và các phòng xét nghiệm vệ tinh

Các câu hỏi liên quan đến việc sử dụng và mục tiêu của xét nghiệm nhanh tại chỗ và các thiết bị.

2.1 Có chương trình xét nghiệm nhanh tại chỗ hoặc xét nghiệm tại giường?

( ) Có ( ) Không

2.2 Có giám đốc chương trình xét nghiệm nhanh tại chỗ không?

( ) Có ( ) Không

2.3 Có một điều phối viên xét nghiệm nhanh tại chỗ, có nghĩa là, nhân viên quản lý và giám sát chương trình POCT?

( ) Có ( ) Không

2.4 Liệt kê các xét nghiệm được thực hiện tại giường bệnh nhân hoặc gần người bệnh ở các khoa phòng như sau:

Bệnh phòng ....

Phòng mổ ....

Phòng cấp cứu ....

Phòng sinh ....

ICU...

Đơn vị chăm sóc tích cực sơ sinh ....

Đơn vị chăm sóc tim mạch ....

Khác ....

2.5 Xác định các xét nghiệm nhanh tại chỗ hoặc các thiết bị bệnh viện của bạn KHÔNG CÓ, nhưng muốn thực hiện trong các khoa phòng như sau:

Bệnh phòng...

Phòng mổ ....
Phòng cấp cứu ....
Phòng sinh ....
ICU..
Đơn vị chăm sóc tích cực sơ sinh ....
Đơn vị chăm sóc tim mạch..
Khác ....

2.6. Xin vui lòng xếp hạng các xét nghiệm nhanh tại chỗ mà bạn cần nhiều nhất và nơi đặt các xét nghiệm chẩn đoán (ví dụ như nhà bệnh nhân, nơi chăm sóc ban đầu, phòng khám, trung tâm y tế dự phòng và nâng cao sức khỏe, phòng cấp cứu, phòng sinh, đơn vị chăm sóc tích cực, khu điều trị chung, hoặc xe cứu thương).

**Xét nghiệm chẩn đoán**	**Xếp Hạng**	**Nơi đặt**
Nhóm máu (A, B, O, Rh)	_____	_____________
Xét nghiệm vi sinh nhanh	_____	_____________
Đông máu (PT / INR)	_____	_____________
Khí máu (pH, pO_2, PCO_2)	_____	_____________
Sinh hóa / điện giải đồ / canxi ion hóa	_____	_____________
Huyết học (CTM, phân loại bạch cầu, tiểu cầu)	_____	_____________
Chỉ điểm tim mạch (cTnT, cTnI, những xét nghiệm khác)	_____	_____________
Đo oxy theo nhịp đập (Đo độ bão hòa O_2)	_____	_____________

2.7. Đối với các xét nghiệm nhanh tại chỗ, xếp hạng **5 xét nghiệm chẩn đoán** từ hữu ích nhất (1) đến ít nhất hữu ích (5):

1. (hữu ích nhất) _____________ 2. _____________ 3. _____________
4. _____________ 5. _____________

2.8. Xác định và xếp hạng các xét nghiệm nhanh tại chỗ/thiết bị sử dụng thường xuyên nhất ở **khoa cấp cứu.**

1. _____________ 6. _____________
2. _____________ 7. _____________
3. _____________ 8. _____________
4. _____________ 9. _____________
5. _____________ 10. _____________

2.9 Xác định và xếp hạng các xét nghiệm nhanh tại chỗ/thiết bị sử dụng thường xuyên nhất ở **ICU.**

1. _____________ 6. _____________
2. _____________ 7. _____________
3. _____________ 8. _____________
4. _____________ 9. _____________
5. _____________ 10. _____________

2.10 Xác định và xếp hạng các xét nghiệm nhanh tại chỗ/thiết bị sử dụng thường xuyên nhất ở **phòng mổ.**

1. _____________ 6. _____________

2. ______________ 7. ______________
3. ______________ 8. ______________
4. ______________ 9. ______________
5. ______________ 10. ______________

2.11 Xác định và xếp hạng các xét nghiệm nhanh tại chỗ/thiết bị sử dụng thường xuyên nhất ở **NICU.**

1. ______________ 6. ______________
2. ______________ 7. ______________
3. ______________ 8. ______________
4. ______________ 9. ______________
5. ______________ 10. ______________

2.12 Xác định và xếp hạng các xét nghiệm nhanh tại chỗ/thiết bị sử dụng thường xuyên nhất ở **CCU.**

1. ______________ 6. ______________
2. ______________ 7. ______________
3. ______________ 8. ______________
4. ______________ 9. ______________
5. ______________ 10. ______________

2.13 Xác định và xếp hạng các xét nghiệm nhanh tại chỗ/thiết bị sử dụng thường xuyên nhất ở **phòng sinh.**

1. ______________ 6. ______________
2. ______________ 7. ______________
3. ______________ 8. ______________
4. ______________ 9. ______________
5. ______________ 10. ______________

2.14 Xác định và xếp hạng các xét nghiệm nhanh tại chỗ/thiết bị sử dụng thường xuyên nhất ở **bệnh phòng.**

1. ______________ 6. ______________
2. ______________ 7. ______________
3. ______________ 8. ______________
4. ______________ 9. ______________
5. ______________ 10. ______________

2.15 Xác định và xếp hạng các xét nghiệm nhanh tại chỗ/thiết bị sử dụng thường xuyên nhất ở **xe cứu thương.**

1. ______________ 6. ______________
2. ______________ 7. ______________
3. ______________ 8. ______________
4. ______________ 9. ______________
5. ______________ 10. ______________

2.16 Những xét nghiệm nhanh tại chỗ nào mà bạn muốn đặt tại **các trung tâm chăm sóc sức khỏe ban đầu, phòng khám, hoặc trung tâm y tế dự phòng và nâng cao sức khỏe** để tăng cường tiếp cận bệnh nhân để chẩn đoán? Danh sách và xếp hạng:

1. ______________ 6. ______________
2. ______________ 7. ______________

3. _____________ 8. _____________
4. _____________ 9. _____________
5. _____________ 10. ____________

2.17 Loại xét nghiệm nhanh tại chỗ nào bạn sẽ đặt tại **nhà bệnh nhân,** để họ có thể làm theo dõi và gọi, email, hay chuyển kết quả trực tiếp thông qua kết nối Internet cho bạn hay một bác sĩ? Danh sách và xếp hạng:

1. _____________ 6. _____________
2. _____________ 7. _____________
3. _____________ 8. _____________
4. _____________ 9. _____________
5. _____________ 10. ____________

2.18 Giả sử một bệnh nhân đột nhiên ốm nặng ở nhà. Xét nghiệm nhanh tại chỗ nào **Nhân viên y tế cấp cứu** thực hiện trong khi vận chuyển bệnh nhân để tăng tốc độ điều trị. Danh sách và xếp hạng:

1. _____________ 6. _____________
2. _____________ 7. _____________
3. _____________ 8. _____________
4. _____________ 9. _____________
5. _____________ 10. ____________

2.19 Ai chủ yếu thực hiện các xét nghiệm nhanh tại chỗ?

( ) Bác sĩ ( ) Điều dưỡng ( ) Nhân viên phòng XN ( )Khác………..
………………

2.20 Bệnh nhân có tự thực hiện xét nghiệm hoặc theo dõi tại các địa điểm chăm sóc bệnh nhân?

( ) Có ( ) Không

Nếu có, xét nghiệm nào (liệt kê) ....

2.21 Sau khi rời khỏi bệnh viện, bệnh nhân có thực hiện xét nghiệm tại nhà của họ không?

( ) Có ( ) Không

- Nếu có, xét nghiệm đó là gì? Hãy ghi rõ ....

2.22 Bệnh nhân có tham khảo ý kiến bác sĩ về kết quả xét nghiệm tự thực hiện không?

( ) Có ( ) Không

2.23 Bệnh nhân hoặc gia đình họ có được đào tạo về cách tự xét nghiệm không?

( ) Có ( ) Không

2.24 Những loại xét nghiệm nhanh tại chỗ bổ sung nào nên được cung cấp cho bệnh viện của bạn?
Xin hãy chỉ ra cụ thể…………………………………………………………………………

2.25 Những loại xét nghiệm nhanh tại chỗ bổ sung nào cần được cung cấp cho bệnh nhân để tự thực hiện?

Xin hãy chỉ ra cụ thể…………………………………………………………………………

2.26 Để tạo điều kiện cho bệnh nhân tiếp cận dịch vụ chăm sóc sức khỏe, xét nghiệm nhanh tại chỗ nên được đặt ở đâu trong mạng lưới chăm sóc sức khỏe của bạn?

( ) Ở nhà bệnh nhân, cho phép họ tự thực hiện xét nghiệm

( ) Tại một địa điểm chăm sóc ban đầu hoặc trong một trung tâm y học dự phòng và nâng cao sức khỏe, nhà bệnh nhân và nơi làm việc.

( ) Ở bệnh viện gần nơi bệnh nhân sống, và nếu có, cách _______ (km)

( ) Tại một bệnh viện khu vực, và nếu có, ở khoảng cách tối đa từ nhà ____ (km)

( ) Khác, ghi rõ ....

CHỦ ĐỀ III. CHĂM SÓC TÍCH CỰC VÀ TIẾP CẬN BỆNH NHÂN

- **Xe cứu thương**

3.1 Bạn có vận chuyển máy bay trực thăng?

( ) Có, xác định tần số mỗi tháng: .... ( ) Không

3.2 Xe cứu thương của bạn có thể đi bao xa để đón một bệnh nhân?

Hãy xác định khoảng cách .... (km) và vị trí ....

3.3 Nếu bệnh nhân cách 5km, 10 km, 25 km, và 50 km thì khoảng bao lâu xe cứu thương đến đón bệnh nhân và đưa tới bệnh viện?

Khoảng cách	Khoảng thời gian (phút)	Nơi đón thực tế
5 km		
10 km		
25 km		
50 km		

3.4 Nhân viên y tế cấp cứu của bạn vận chuyển bệnh nhân bằng xe cứu thương bao lâu một lần?

.... một ngày .... một tháng .... một năm

3.5 Bệnh viện của bạn có cung cấp cho bệnh nhân địa phương xe cứu thương trong trường hợp khẩn cấp hoặc thiên tai (ví dụ, lũ lụt, động đất, bão)?

( ) Có ( ) Không

3.6 Bạn có cần vận chuyển **bệnh nhân nặng** tới bệnh viện khác?

( ) Có, xác định cự ly vận chuyển ....(km) và nơi giới thiệu đến ..... ( ) Không

Nếu có, tại sao ....

Nếu không, tại sao không ....

3.7 Nếu cần chuyển bệnh nhân sang các bệnh viện khác, bạn chuyển họ đi đâu và mất bao lâu?

**Tên của bệnh viện chuyển tiếp**	**Khoảng thời gian (phút)**

3.8 Xác định các bệnh viện cộng đồng gần nhất với bệnh viện của bạn ....

- Xác định khoảng cách .... km.
- Xác định thời gian đi lại .... .phút.

3.9 Xác định bệnh viện tuyến trên (hoặc bệnh viên khu vực) gần nhất với bệnh viện của bạn ....

- Xác định khoảng cách .... km.
- Xác định thời gian đi lại .... .phút.

3.10 Hãy xác định số lượng các trung tâm chăm sóc sức khỏe ban đầu, phòng khám chăm sóc sức khỏe ban đầu, trung tâm y tế dự phòng và nâng cao sức khỏe (HPH), hoặc các đơn vị chăm sóc ban đầu (PCU) thuộc phạm vi trách nhiệm của bệnh viện bạn:

**Tên**	**Khoảng cách (km)**	**Thời gian đi lại (Phút)**	**Nhân viên**			
			**Bác sĩ**	**Số giờ làm việc**	**Điều dưỡng**	**Số giờ làm việc**
1.						
2.						
3.						
4.						
5.						
6.						
7.						
8.						
9.						
10.						

3.11 Bây giờ mô tả các vị trí xe cứu thương bao phủ từ bệnh viện của bạn. Khoảng cách, thời gian vận chuyển và điểm đến.

<u>**Vị trí (đón)**</u> <u>**Khoảng cách (km)**</u> <u>**Thời gian vận chuyển (phút)**</u> <u>**Điểm đến**</u>

1. ______________ → ______________ → ______________ → __________
2. ______________ → ______________ → ______________ → __________
3. ______________ → ______________ → ______________ → __________
4. ______________ → ______________ → ______________ → __________

→ →

→ →

→

5. ____________ → ____________   ____________   ____________
6. ____________ → ____________   ____________   ____________
7. ____________ → ____________   ____________ → ____________
8. ____________ → ____________ → ____________ → ____________
9. ____________ → ____________ → ____________ → ____________
10. ____________ → ____________ → ____________ → ____________

3.12 Có bao nhiêu bệnh nhân đến bệnh viện của bạn mỗi ngày? ....

Tỷ lệ phần trăm bị bệnh cấp tính? ....%

Tỷ lệ phần trăm bệnh mạn tính?....... ....%

- **Các trường hợp bệnh tim mạch (ví dụ, nhồi máu cơ tim cấp tính)**

3.13 Cơ sở y tế mà bạn tương tác cho việc chăm sóc tim mạch? Vui lòng điền vào các chi tiết bên dưới. [CH, bệnh viện cộng đồng; RH, bệnh viện khu vực]

**Tên**	**Loại (CH, RH, loại khác)**	**Khoảng cách (Km)**	**Thời gian (Phút)**

3.14 5 thiết bị và/hoặc công nghệ hàng đầu mà bạn muốn có để hỗ trợ chẩn đoán bệnh tim mạch là gì?

**Thứ tự**	**Miêu tả**
1	
2	
3	
4	
5	

3.15 Bạn có các bác sĩ chuyên khoa chăm sóc tim mạch, chẳng hạn như tim mạch can thiệp?

Nếu có, hãy mô tả: ....

3.16 Xét nghiệm chẩn đoán nào bạn sử dụng để hỗ trợ việc chẩn đoán nhồi máu cơ tim cấp tính (AMI)? ( ) cTnI ( ) cTnT ( ) Các chỉ điểm sinh học khác: ....

3.17 Nếu bạn chẩn đoán dương tính với nhồi máu cơ tim cấp (AMI), bạn có thể nhanh chóng cung cấp dịch vụ chăm sóc can thiệp?

( ) Có, thời gian đáp ứng: .... ( ) Không

- Nếu không, nơi họ sẽ đi để được chăm sóc?

+ Khoảng cách đi lại (km)? _____

+ Thời gian đi lại (phút)? _____

3.18 Các bước xử trí tiếp theo là gì nếu chẩn đoán dương tính với các bệnh/tình trạng sau đây?

**Bệnh / Tình trạng**	**Bước tiếp theo**
Nhồi máu cơ tim cấp tính	
Chấn thương cấp tính	
Phẫu thuật cấp tính	
Bệnh đái tháo đường (ĐTĐ nhiễm toan ceton)	
Tăng huyết áp	
Đột quỵ	
Hen suyễn	
Nhiễm trùng huyết	
Triệu chứng hô hấp	
Triệu chứng tiêu hóa	
Rối loạn niệu đạo & đường tiết niệu	
Bệnh nhiễm trùng	
HIV / bệnh lây truyền qua đường tình dục	
Khác)……………………………………………	

3.19 Nếu các bước tiếp theo là chuyển đến một bệnh viện khác để được chăm sóc, bệnh viện nào bạn chuyển, mất bao lâu, và bao xa?

**Bệnh / Tình trạng**	**Bệnh viện tuyến trên chuyển tiếp**	**Thời gian (phút)**	**Khoảng cách (km)**
Nhồi máu cơ tim cấp tính			
Chấn thương cấp tính			
Phẫu thuật cấp tính			
Bệnh đái tháo đường (ĐTĐ nhiễm toan ceton)			
Tăng huyết áp			
Đột quỵ			
Hen suyễn			
Nhiễm trùng huyết			
Triệu chứng hô hấp			
Triệu chứng tiêu hóa			
Rối loạn niệu đạo & đường tiết niệu			
Bệnh truyền nhiễm			
HIV / bệnh lây truyền qua đường tình dục			
Khác)……………………………………………			

- **Đái tháo đường**

3.20 Xét nghiệm sàng lọc glucose được thực hiện ở đâu?

( ) Nhà ( ) Cộng đồng địa phương ( ) Trạm y tế xã gần nhất

( ) Trung tâm y tế dự phòng và nâng cao sức khỏe hoặc đơn vị chăm sóc ban đầu

( ) Khác ....

3.21 Ai làm xét nghiệm sàng lọc glucose?

( ) Bác sĩ ( ) Điều dưỡng ( ) NV y tế công cộng ( ) Bệnh nhân

( ) Khác, ghi rõ..

3.22 Loại mẫu được sử dụng để thử nghiệm glucose?

( ) Máu mao mạch ( ) Huyết tương ( ) Huyết thanh ( ) Khác ....

3,23 Ai giải thích kết quả của xét nghiệm glucose?

( ) Bác sĩ ( ) Điều dưỡng ( ) NV sức khỏe cộng đồng ( ) Bệnh nhân

( ) Khác ....

3.24 Điểm cắt tính bằng mmol/L bạn sử dụng cho **Tiền Đái tháo đường?**

Từ .... mmol/L đến .... mmol/L

3,25 Điểm cắt tính bằng mmol/L bạn sử dụng cho **Đái tháo đường?**

Từ .... mmol/L đến .... mmol/L

3.26 Có một chương trình sàng lọc bệnh đái tháo đường trong một cộng đồng không?

( ) Có ( ) Không

- Nếu vậy, xét nghiệm sàng lọc được thực hiện ở đâu?

( ) Cộng đồng địa phương ( ) Trạm y tế xã gần nhất

( ) Trung tâm y tế dự phòng và nâng cao sức khỏe ( ) Khác ....

3.27 Bệnh nhân có sử dụng máy đo glucose để tự theo dõi nồng độ glucose trong máu?

( ) Có ( ) Không

Nếu vậy, ai trả tiền cho máy đo đường huyết? Xin hãy chỉ ra cụ thể………………

3.28 Nếu bệnh nhân có biến chứng (ví dụ, suy thận, bệnh võng mạc), nơi bệnh nhân được điều trị? Xin hãy chỉ ra cụ thể……………………………

- **Xét nghiệm hỗ trợ chẩn đoán**

3.29 Đối với mỗi một trong các bệnh/tình trạng sau xét nghiệm gì được thực hiện để chẩn đoán? Hãy điền các xét nghiệm cho các bệnh/ tình trạng sau

Bệnh / Tình trạng	Xét nghiệm chẩn đoán
Nhồi máu cơ tim cấp tính	
Chấn thương cấp tính	
Phẫu thuật cấp tính	
Bệnh đái tháo đường (ĐTĐ nhiễm toan ceton)	
Tăng huyết áp	
Đột quỵ	
Hen suyễn	
Nhiễm trùng huyết	
Triệu chứng hô hấp	
Triệu chứng tiêu hóa	
Rối loạn niệu đạo & đường tiết niệu	
Bệnh nhiễm trùng	
HIV / bệnh lây truyền qua đường tình dục	
Khác)……………………………………………….	

3.30 Ở bệnh viện nào bạn sẽ tìm thấy loại hỗ trợ này?

Bệnh / Tình trạng	Tên bệnh viện
Nhồi máu cơ tim cấp tính	
Chấn thương cấp tính	
Phẫu thuật cấp tính	
Bệnh đái tháo đường (ĐTĐ nhiễm toan ceton)	
Tăng huyết áp	
Đột quỵ	
Hen suyễn	
Nhiễm trùng huyết	
Triệu chứng hô hấp	
Triệu chứng tiêu hóa	
Rối loạn niệu đạo & đường tiết niệu	
Bệnh nhiễm trùng	
HIV / bệnh lây truyền qua đường tình dục	
Khác)……………………………………………….	

3,31 Loại thiết bị xét nghiệm nhanh tại chỗ hoặc bộ dụng cụ xét nghiệm nào hiện có ở BV bạn?

Bệnh / Tình trạng	Thiết bị POC / bộ thử
Nhồi máu cơ tim cấp tính	
Chấn thương cấp tính	
Phẫu thuật cấp tính	
Bệnh đái tháo đường (ĐTĐ nhiễm toan ceton)	
Tăng huyết áp	
Đột quỵ	
Hen suyễn	
Nhiễm trùng huyết	
Triệu chứng hô hấp	
Triệu chứng tiêu hóa	
Rối loạn niệu đạo & đường tiết niệu	
Bệnh nhiễm trùng	
HIV / bệnh lây truyền qua đường tình dục	
Khác)…………………………………………….	

3,32 Trong mạng lưới chăm sóc sức khỏe của bạn, nếu bệnh viện của bạn thiếu xét nghiệm chẩn đoán quan trọng trong trường hợp khẩn cấp hoặc thiên tai thì bạn thường tìm kiếm chỗ nào?

Xin hãy chỉ ra cụ thể…………………………………………………………………….

- **Chăm sóc đặc biệt**

3.33 Các cơ sở y tế nào mà bệnh viện của bạn hỗ trợ nếu cơ sở bạn đóng vai trò như một bệnh viện chuyển tiếp?

Xin hãy chỉ ra cụ thể……………………………………………………………………..

....

....

3.34 Các cơ sở y tế nào mà bệnh viện của bạn cần tìm kiếm các chăm sóc y tế chuyên sâu?

Chăm sóc y tế chuyên sâu	Cơ sở y tế

3.35 Trong mạng sức lưới chăm sóc sức khỏe của bạn, làm thế nào bạn cải thiện việc tiếp cận để chăm sóc cho bệnh nhân ngừng tim?

Vui lòng giải thích………………………………………………………………………

....

CHỦ ĐỀ IV. XÉT NGHIỆM LÂM SÀNG

- **Thời gian dịch vụ**

4.1 Số ngày làm việc trong tuần Thời gian: .... .Đến ....

Cuối tuần / ngày lễ Thời gian: .... .Đến ....

Dịch vụ trực trực tuyến ( ) Có ( ) Không

4.2 Hãy liệt kê và xếp hạng hai mươi **xét nghiệm chẩn đoán** mà bạn cần nhất để chăm sóc cho bệnh nhân của bạn (1, quan trọng nhất; 20, ít quan trọng nhất):

Quan trọng nhất

1. __________ 2. __________ 3. __________ 4. __________ 5. __________

6. __________ 7. __________ 8. __________ 9. __________ 10. _________

11. _________ 12. _________ 13. _________ 14. __________ 15. _________

16. _________ 17. _________ 18. _________ 19. __________ 20. _________ *Ít quan trọng nhất*

4.3 Hãy xếp hạng các **bộ phận phòng xét nghiệm** sau đây theo thứ tự tầm quan trọng (1, cao nhất):

_____ Sinh hóa _____Chẩn đoán phân tử

_____ Huyết học _____ Tế bào học

_____ Vi sinh học _____ Theo dõi không xâm lấn (máy đo oxy theo nhịp đập, đo độ bão hòa oxy)

_____ Miễn dịch học _____ khác (ghi rõ tên bộ phận): ____________

_____ Kính hiển vi _____ Khác (ghi rõ): __________________________

_____ Ngân hàng máu _____ Khác (ghi rõ): __________________________

- **Số bệnh nhân, địa điểm, và tiếp cận chăm sóc sức khỏe**

4.4 Bạn khám bao nhiêu bệnh nhân mỗi ngày / tháng / năm?

.... một ngày .... một tháng .... một năm

4.5 BN đến từ đâu nhiều nhất? Vui lòng chỉ ra vị trí ....

4.6 Hầu hết bệnh nhân đến cơ sở của bạn như thế nào?

Hãy nêu rõ những điều sau:

.... % Đi bộ

.... % Xe máy

.... % Ô tô

.... % Phương tiện công cộng

.... % Xe cứu thương

.... % Máy bay trực thăng

4.7 Xin vui lòng cung cấp số lần thăm khám ước lượng cho các bệnh / tình trạng sau

**Bệnh / tình trạng**	**Số lần thăm khám ước tính**
Nhồi máu cơ tim cấp tính	
Chấn thương cấp tính	
Phẫu thuật cấp tính	
Bệnh đái tháo đường (ĐTĐ nhiễm toan ceton)	
Tăng huyết áp	
Đột quỵ	
Hen suyễn	
Nhiễm trùng huyết	
Triệu chứng hô hấp	
Triệu chứng tiêu hóa	
Rối loạn niệu đạo & đường tiết niệu	
Bệnh nhiễm trùng	
HIV / bệnh lây truyền qua đường tình dục	
Khác)………………………………………………	

- **Theo dõi độ bão hòa oxy (Máy đo oxy bằng nhịp đập)**

4.8 Có bất kỳ thiết bị theo dõi độ bão hòa oxy nào không (Máy đo oxy bằng nhịp đập)?

( ) Có ( ) Không

Nếu **có, hãy** xác định số lượng máy đo độ bão hòa oxy ....

4.9 Xác định số lượng máy đo độ bão hòa oxy có sẵn trong mỗi khoa phòng:

Cấp cứu ....

Phòng mổ...............................

ICU ....

NICU ....

CCU ....

Phòng sinh ....

Bệnh phòng ....

Xe cứu thương...................

Khác ....

- **Chăm sóc tích cực**

4.10 Xét nghiệm khí máu được thực hiện trong phòng xét nghiệm?

( ) Có ( ) Không

Nếu có, các phép đo sau đây được thực hiện trong phòng xét nghiệm?

pO_2	( ) Có	( ) Không
PCO_2	( ) Có	( ) Không
pH	( ) Có	( ) Không
HCO_3	( ) Có	( ) Không

4.11 Điện giải đồ được thực hiện tại phòng xét nghiệm?

( ) Có ( ) Không

- Nếu vậy, xin ghi rõ những chất điện giải đồ ....

4.12 Ai thực hiện các xét nghiệm sau đây?

- Khí máu ( ) BS ( ) Điều dưỡng ( ) Nhân viên phòng xét nghiệm
  ( ) Khác ....
- Điện giải đồ ( ) BS ( ) Điều dưỡng ( ) Nhân viên phòng xét nghiệm
  ( ) Khác ....
- Canxi ion hóa ( ) BS ( ) Điều dưỡng ( ) Nhân viên phòng xét nghiệm
  ( ) Khác ....
- PO_2 ( ) BS ( ) Điều dưỡng ( ) Nhân viên phòng xét nghiệm
  ( ) Khác ....
- PCO_2 ( ) BS ( ) Điều dưỡng ( ) Nhân viên phòng xét nghiệm
  ( ) Khác ....
- PH ( ) BS ( ) Điều dưỡng ( ) Nhân viên phòng xét nghiệm
  ( ) Khác ....
- HCO_3 ( ) BS ( ) Điều dưỡng ( ) Nhân viên phòng xét nghiệm
  ( ) Khác ....

4.13 Có phòng xét nghiệm thực hiện cấy máu?

( ) Có ( ) Không

4.14 Phòng xét nghiệm nào và ở đâu các xét nghiệm sau được thực hiện?

- Viêm gan A ( ) Có ( ) Không Ở đâu ....
- Viêm gan B (HBsAg) ( ) Có ( ) Không Ở đâu ....

- Viêm gan B (HB Ab) ( ) Có ( ) Không Ở đâu ....
- Bệnh cúm A ( ) Có ( ) Không Ở đâu ....
- Bệnh cúm B ( ) Có ( ) Không Ở đâu ....
- H1N1 ( ) Có ( ) Không Ở đâu ....
- H7N9 ( ) Có ( ) Không Ở đâu ....
- Mers-CoV ( ) Có ( ) Không Ở đâu ....
- HIV (AIDS) ( ) Có ( ) Không Ở đâu ....
- Nhiễm liên cầu ( ) Có ( ) Không Ở đâu ....
- Cấy máu ( ) Có ( ) Không Ở đâu ....
- Mọc trên đĩa nuôi cấy ( ) Có ( ) Không Ở đâu ....

4.15 Trừ các xét nghiệm trong mục **4.14,** có xét nghiệm nào khác cho các bệnh nhiễm trùng?

....

....

- **Bệnh tim mạch**

4.16 Xét nghiệm nào sau đây được thực hiện?

- cTn I (cardiac troponin I) ( ) Có ( ) Không

  Nếu vậy, loại: ( ) định tính ( ) định lượng
- cTn T (c ardiac troponin T) ( ) Có ( ) Không

  Nếu vậy, loại: ( ) định tính ( ) định lượng
- CK ( ) Có ( ) Không
- CK-MB ( ) Có ( ) Không
- LD (LDH) ( ) Có ( ) Không
- AST ( ) Có ( ) Không
- Myoglobin ( ) Có ( ) Không
- BNP hoặc NT-pro BNP ( ) Có ( ) Không
- Khác ....

- **Huyết học**

4.17 Có xét nghiệm công thức máu?

( ) Có ( ) Không

4.18 Có xét nghiệm đông máu?

( ) Có ( ) Không

- Nếu vậy, những loại xét nghiệm nào được thực hiện trong phòng xét nghiệm?

  ( ) PT ( ) APTT ( ) PT và APTT ( ) Khác...........................

4.19 Các điều dưỡng có thực hiện bất kỳ xét nghiệm cận lâm sàng giống như nhân viên phòng xét nghiệm không?

( ) Có ( ) Không

- Nếu có, xin ghi rõ các xét nghiệm cận lâm sàng mà Điều dưỡng có thể thực hiện

  ( ) Huyết học, ghi rõ ....

  ( ) Huyết thanh học, ghi rõ ....

  ( ) Vi sinh, ghi rõ ....

  ( ) Sinh hóa, ghi rõ ....

  ( ) Xét nghiệm nước tiểu, ghi rõ ....

  ( ) Khác, ghi rõ ....

4.20 Bất kỳ thiết bị hoặc xét nghiệm mới mà phòng xét nghiệm bạn vẫn cần?

....

4.21 Xét nghiệm nào mà phòng xét nghiệm của bạn cần phải gửi đến phòng xét nghiệm bên ngoài hoặc tại phòng xét nghiệm tuyến trên?

....

4.22 Hãy xác định tên và vị trí của phòng xét nghiệm bên ngoài hoặc tại phòng xét nghiệm tuyến trên?

....

4.23 Bao lâu để nhận được kết quả xét nghiệm từ phòng xét nghiệm bên ngoài hoặc tại phòng xét nghiệm tuyến trên? (Xác định thời gian quay vòng)

....

4.24 Những loại xét nghiệm nào được thực hiện ở các trung tâm y tế dự phòng và nâng cao sức khỏe?

( ) Glucose ( ) Mang thai ( ) Nước tiểu

( ) Protein ( ) Hematocrit ( ) Khác ....

4.25 Các thiết bị hoặc xét nghiệm mới nào sẽ giúp cải thiện kết quả sức khỏe của bệnh nhân tại các trung tâm y tế dự phòng và nâng cao sức khỏe?

....

....

4.26 Ai chỉ định các xét nghiệm chẩn đoán tại trung tâm y tế dự phòng và nâng cao sức khỏe?

( ) BS ( ) Điều dưỡng ( ) Nhân viên phòng XN ( ) Cán bộ y tế công cộng

( ) Khác..................................

4.27 Ai chịu trách nhiệm thực hiện quản lý chất lượng của các xét nghiệm chẩn đoán tại trung tâm y tế dự phòng và nâng cao sức khỏe?

( ) BS ( ) Điều dưỡng ( ) Nhân viên phòng XN ( ) Cán bộ y tế công cộng

( ) Khác..................................

CHỦ ĐỀ V. GIẢI QUYẾT VẤN ĐỀ Y TẾ Ở CỘNG ĐỒNG

5.1 Xin liệt kê và xếp loại các vấn đề y khoa phổ biến nhất trong cộng đồng của bạn (1, đầu trang, 10, ít quan trọng nhất)

1 .... 6. ....

2 .... 7. ....

3 .... 8. ……………………………..

4……………………………9. ....

5 .... 10. ....

5.2 Những vấn đề y tế nào bị lãng quên?

Xin hãy chỉ ra cụ thể……………………………………………………………………………

5.3 Những vấn đề y tế nào ảnh hưởng nhiều nhất đến hoạt động của người dân?

Xin hãy chỉ ra cụ thể……………………………………………………………………………

5.4 Trong trường hợp khẩn cấp, nơi nào người dân được chuyển để nhận được dịch vụ chăm sóc sức khoẻ?

Xin hãy chỉ ra cụ thể……………………………………………………………………………

5.5 Các chẩn đoán phổ biến nhất mà bạn không thể điều trị tại bệnh viện của mình và do đó, phải chuyển đến bệnh viện khác? Liệt kê và xếp hạng: (1, quan trọng nhất, 5, ít quan trọng nhất)

1 .... (Phổ biến nhất)

2 ....

3 ....

4 ....

5 .... (Ít phổ biến nhất)

5.6 Dành cho người khảo sát điền vào: Xác định những **cải tiến có tác động quan trọng nhất và cao nhất** khi thực hiện xét nghiệm nhanh tại chỗ sẽ tạo ra tại nơi này, dựa trên bằng chứng khảo sát thực tế.

1. ....

....

....

2 ....

....

....

3 ....

....

....

Phiên bản 10.4

Ngày 7 tháng 7 năm 2017
