# Supplementary material for: Optimizing Public Health Preparedness for Highly Infectious Diseases in Central Vietnam
Source: Diagnostics (Basel). 2022 Aug 24;12(9):2047. doi: 10.3390/diagnostics12092047 (PMC9497681; doi:10.3390/diagnostics12092047)
Supplement: Supplementary file 1 [file diagnostics-12-02047-s001.zip › S4-Infectious Disease Survey Questionnaire.pdf]

TOPIC VI: Infectious Disease Survey Questionnaire

6.1 Does your hospital have preparations in place for highly infectious disease outbreaks/epidemics?

6.2 If a patient is suspected of having Ebola, is the hospital prepared? (yes/no)

6.3 Does your hospital have the diagnostic tests necessary to detect Ebola?

6.4 Does your hospital have PPE (personal protective equipment) available for staff when dealing with patients suspected of having a highly infectious disease (e.g. Ebola)?

6.5 Are staff trained to use PPE? (yes/no) If yes, which staff members: doctors, nurses, lab staff?

6.6 Is there an isolation unit or facility outside/near the main hospital for patients suspected of having a highly infectious disease?

6.7 Is there an Isolation laboratory available for patients suspected of having a highly infectious agent?

6.8 If a patient walks in with Ebola Virus Disease (EVD), what are the first steps taken to **isolate/quarantine** the patient with EVD?

6.9 Is the patient transferred to a referral hospital?

6.10 What method of transportation is used?

6.11 If a patient walks in with Ebola Virus Disease (EVD), what are the first steps taken to **detect** and diagnose EVD?

6.12 Do you have molecular diagnostics available for any of the highly infectious diseases below? (yes/no) If yes, please specify.

Zika Virus:
Mers-CoV:
Ebola:
Avian Flu:
SARS:
TB:
Resistant TB:
Others:

6.13 In general, does your hospital have the proper isolation/quarantine equipment and facility to successfully contain a highly infectious disease? If yes, please explain.

6.14 In general, does your hospital have molecular diagnostics available to detect for highly infectious diseases? If yes, please specify.
