# Supplementary material for: Optimizing Public Health Preparedness for Highly Infectious Diseases in Central Vietnam
Source: Diagnostics (Basel). 2022 Aug 24;12(9):2047. doi: 10.3390/diagnostics12092047 (PMC9497681; doi:10.3390/diagnostics12092047)
Supplement: Supplementary file 1 [file diagnostics-12-02047-s001.zip › S5-Bộ Câu Hỏi Khảo Sát Về Bệnh Truyền Nhiễm.pdf]

## CHỦ ĐỀ VI: BỘ CÂU HỎI KHẢO SÁT BỆNH TRUYỀN NHIỄM

**6.1** Bệnh viện của bạn có chuẩn bị sẵn nơi cách li trong trường hợp bùng phát bệnh dịch không?

**6.2** Bệnh viện có chuẩn bị sẵn sàng nếu một bệnh nhân bị nghi ngờ nhiễm Ebola nhập viện không? (có hoặc không)

**6.3** Bệnh viện của bạn có các xét nghiệm chẩn đoán cần thiết để phát hiện Ebola không?

**6.4** Bệnh viện của bạn có sẵn sẵn các thiết bị phòng hộ cá nhân cho nhân viên y tế khi tiếp xúc với những bệnh nhân mắc các bệnh truyền nhiễm nặng (ví dụ: Ebola) hay không?

**6.5** Các nhân viên y tế có được đào tạo cách sử dụng các thiết bị phòng hộ cá nhân không? (có/không) Niếu có, đó là bộ phận nhân viên nào: bác sĩ, điều dưỡng, nhân viên phòng xét nghiệm?

**6.6** Có các đơn vị cách li hoặc cơ sở bên ngoài/ nằm gần bệnh viện chính cho các bệnh nhân nghi ngờ mắc các bệnh truyền nhiễm nặng không?

**6.7**
Có phòng xét nghiệm cách li sẵn trong trường hợp có bệnh nhân nghi ngờ nhiễm các tác nhân lây truyền mạnh hay không?

**6.8** Trong trường hợp có một bệnh nhân nhập viện do nhiễm Ebola thì bước xử lí đầu tiên để cách li bệnh nhân này là gì?

**6.9** Bệnh nhân có được chuyển lên bệnh viện tuyến trên hay không?

**6.10** Phương tiện vận chuyển bệnh nhân là gì?

**6.11** Trong trường hợp một bệnh nhân nhập viện do nhiễm Ebola thì bước xử lí đầu tiên để phát hiện và chẩn đoán bệnh nhiễm virus Ebola là gì?

**6.12** Bệnh viện có sẵn những xét nghiệm phân tử để chẩn đoán các bệnh truyền nhiễm sau hay không? (có/không) Nếu có, nêu rõ là xét nghiệm gì?

Vi rút Zika:
Mers-CoV:
Ebola:
Cúm gia cầm:
SARS:
Lao:
Lao đề kháng:
Khác:

**6.13** Nhìn chung, bệnh viện của bạn có các thiết bị và các cơ sở cách li phù hợp để cách li các bệnh truyền nhiễm nặng hay không? Nếu có, vui lòng giai thích rõ.

**6.14** Nhìn chung, bệnh viện của bạn có sẵn các xét nghiệm chẩn đoán phân tử để phát hiện các bệnh truyền nhiễm nặng hay không? Nếu có, vui lòng nêu rõ.
